# Augmenting precision medicine via targeted RNA-Seq detection of expressed mutations

**DOI:** 10.1038/s41698-025-00993-8

**Published:** 2025-06-13

**Authors:** Dan Li, Jianying Li, Donald J. Johann, Daniel Butler, Guangchun Chen, Jonathan Foox, Binsheng Gong, Wendell Jones, David P. Kreil, Rebecca Kusko, Paweł P. Łabaj, Anne Bergstrom Lucas, Christopher E. Mason, Christopher Mozsary, Natalia Novoradovskaya, Carlos Pabón-Peña, Bohu Pan, Todd A. Richmond, Roberta Maestro, Sayed Mohammad Ebrahim Sahraeian, Andreas Scherer, Hagen U. Tilgner, James C. Willey, Pierre R. Bushel, Joshua Xu

**Affiliations:** 1 Division of Bioinformatics and Biostatistics, National Center for Toxicological Research, US Food and Drug Administration, Jefferson, AR USA; 2 Integrative Bioinformatics Support Group, Biostatistics and Computational Biology Branch, National Institute of Environmental Health Sciences, Research Triangle Park, Durham, NC USA; 3 Kelly Government Solutions, Inc., Research Triangle Park, Durham, NC USA; 4 Winthrop P Rockefeller Cancer Institute, University of Arkansas for Medical Sciences, Little Rock, AR USA; 5 Department of Physiology and Biophysics, Weill Cornell Medicine, Cornell University, New York, NY USA; 6 Department of Immunology, Genomics and Microarray Core Facility, University of Texas Southwestern Medical Center, Dallas, TX USA; 7 IQVIA Laboratories, Durham, NC USA; 8 Boku University Vienna, Vienna, Austria; 9 Cellino Biotech, Cambridge, MA USA; 10 Małopolska Centre of Biotechnology, Jagiellonian University, Krakow, Poland; 11 Department of Biotechnology, Boku University, Vienna, Austria; 12 Agilent Technologies, Santa Clara, CA USA; 13 Agilent Technologies, La Jolla, CA USA; 14 Computational Biology and Molecular Lab Applications, Roche Sequencing Solutions Inc., Pleasanton, CA USA; 15 Unit of Oncogenetics and Functional Oncogenomics, Centro di Riferimento Oncologico di Aviano (CRO Aviano) IRCCS, Aviano (PN), Italy; 16 EATRIS ERIC, Amsterdam, The Netherlands; 17 Roche Sequencing Solutions, Santa Clara, CA USA; 18 Institute for Molecular Medicine Finland (FIMM), University of Helsinki, Helsinki, Finland; 19 Brain and Mind Research Institute, Weill Cornell Medicine, New York, NY USA; 20 Center for Neurogenetics, Weill Cornell Medicine, New York, NY USA; 21 Departments of Medicine, Pathology, and Cancer Biology, College of Medicine and Life Sciences, University of Toledo Health Sciences Campus, Toledo, OH USA; 22 Biostatistics and Computational Biology Branch, National Institute of Environmental Health Sciences, Research Triangle Park, Durham, NC USA

**Keywords:** Oncology, Genomic analysis, High-throughput screening, Sequencing, Cancer genomics

## Abstract

In precision medicine, DNA-based assays are currently necessary but not always sufficient for predicting therapeutic efficacy of cancer drugs based on the mutational findings in a patient’s tumor specimen. Most drugs target proteins, but it is challenging and not yet cost-effective to perform high-throughput proteomics profiling, including mutational analysis, on cancer specimens. RNA may be an effective mediator for bridging the “DNA to protein divide” and provide more clarity and therapeutic predictability for precision oncology. While RNA sequencing (RNA-seq) has been increasingly used alongside DNA cancer mutation screening panels to assess the impact of variants on gene transcript expression and splicing, comprehensive evaluations of RNA panels and the integration of expressed mutation data analytics to supplement DNA panels are still limited. In this study, we conducted targeted RNA-seq on a reference sample set for expressed variant detection to explore its potential capability to complement DNA variant results or detect variants independently. The results indicated that, with a carefully controlled false positive rate ensuring high accuracy, RNA-seq uniquely identified variants with significant pathological relevance that were missed by DNA-seq, demonstrating its potential to uncover clinically actionable mutations. On the other hand, while some variants were detected by both approaches, others were missed by one or the other, reflecting either the nature of these variants or limitations of the bioinformatics tools used. Variants missed by RNA-seq are often not expressed or expressed at very low levels, suggesting they may be of lower clinical relevance. Incorporating RNA-seq into clinical biomarker panels will ultimately advance precision medicine and improve patient outcomes by improving the strength and reliability of somatic mutation findings for clinical diagnosis, prognosis and prediction of therapeutic efficacy.

## Introduction

Cancer is a genetic disease at the cellular level and is driven by specific genetic variations that impact protein function^1,2^. Accurate profiling of these genetic mutations improves clinical diagnosis, prognosis, and therapeutic efficacy by revealing the unique molecular basis of a patient’s malignancy^3,4^. While panel-based DNA sequencing (DNA-seq) and whole-exome sequencing (WES) are the current accepted standard methods for detecting mutations in tumor samples, they primarily determine the presence or absence of variants without revealing their functional consequence.

DNA may be considered as “potential” since the critical transformative steps of transcription and translation must occur prior to building cellular components and machinery. Proteins mediate biological functions. DNA mutations (e.g., point mutations, insertions, and deletions [indels]) can be detected, measured, and reported with accuracy and precision in a high-throughput, cost-effective manner, greatly aiding individualized cancer care and clinical decision-making. While proteins can be measured, detecting mutations within them is difficult to achieve in a high-throughput, cost effective way. RNA may be an effective mediator for bridging this gap and providing greater clarity and predictability for precision medicine.

RNA sequencing (RNA-seq) provides additional information than DNA-seq, such as whether a variant is expressed and to what level. Thus, RNA-seq bridges the gap between DNA alterations and protein expression activity^5–7^. Furthermore, RNA-seq facilitates the detection of transcript variants, such as alternative splicing and fusion transcripts, which can alter protein function and drive disease phenotypes^8–10^. Additionally, by analyzing non-coding transcript regions, RNA-seq can also identify variants that affect regulatory elements critical for gene expression^11,12^. The comprehensive, novel, and orthogonal insights offered by RNA-seq are thus invaluable, potentially improving precision medicine by strengthening the robustness and predictability of the molecular data, which greatly aids associated clinical decision making.

RNA-seq can often provide a stronger mutation signal for variant detection in moderate to highly expressed genes as the variant allele will be adequately represented in the sequencing data. For instance, Wilkerson et al. demonstrated that integrating RNA-seq and WES data enhanced mutation detection capacity, especially in the case of low-purity tumor samples^5^. Similarly, another study comparing paired RNA-seq and WES for mutation analysis found that RNA-seq revealed more potential novel somatic mutations than WES alone, suggesting that RNA-seq enhances the robustness of somatic mutation identification^6^. Additionally, Rabizadeh and colleagues used RNA-seq to identify tumor somatic single nucleotide variants (SNVs) in lung and other cancers, revealing that up to 18% of these SNVs were not transcribed and indicating that some mutations detected by DNA-seq are likely clinically irrelevant^7^. This highlights the critical need to validate the clinical relevance of mutations using RNA-seq, ensuring that tumor molecular classification and treatment decisions are based on actionable genetic targets, which are known to be expressed in the patient’s tumor.

Despite the advantages mentioned above, RNA-seq faces significant challenges in verifying somatic mutations. These include alignment errors near splice junctions, especially for novel junctions^13^, which can distort findings. Additionally, RNA editing sites might be misinterpreted as DNA genetic variants^14,15^, and variability in gene expression levels can lead to non-uniform read depth. Furthermore, sequencing reads may be disproportionately dominated by highly expressed clinically irrelevant genes (e.g., housekeeping genes)^16^. To overcome these limitations, targeted RNA-seq offers deeper coverage of genes with potential somatic mutations of interest. Thus, higher detection accuracy and more reliable variant identification, especially for rare alleles as well as low-abundant evolving mutant clones, are advantages of targeted RNA-seq^17^. Notably, some targeted RNA-seq panels have been developed for detecting expressed variants. Among these, the Afirma Xpression Atlas (XA) panel, which includes 593 genes covering 905 variants, is available in the United States. and internationally for clinical decision making involving thyroid malignancy^18^. Interestingly, the XA panel has revealed that some DNA variants were poorly detected in traditional bulk RNA-seq due to low expression of the mutated transcript, highlighting the importance of targeted approaches in the management of thyroid cancer^18^.

In real-world practice, it is crucial to consider how best to integrate RNA-seq with DNA-seq to optimize variant detection and accuracy. When DNA-seq is available, it serves as a valuable baseline due to its high accuracy, sensitivity, and relatively low cost that continues to decline. In such cases, RNA-seq can be employed to verify and prioritize the variants detected by DNA-seq, leveraging its ability to confirm their expression and functional relevance of these variants. This integrative strategy not only improves the detection of expressed variants but also serves as a critical step in the development of advanced therapeutic approaches. For example, mRNA-4157 (V940) is a novel mRNA-based individualized neoantigen therapy encoding up to 34 neoantigens, designed to target a patient’s unique set of cancer neoantigens. A neoantigen selection algorithm was developed to verify and prioritize the amino acid candidates^19^. This complementary approach ensures that the clinical implications of somatic mutations are accurately assessed, enhancing the overall precision of genetic diagnostics.

Conversely, in scenarios where DNA-seq is not available, RNA-seq must be analyzed thoughtfully to ensure the reliability of variant detection. This is especially important given the heterogeneity of solid tumors and corresponding variability or “regional” nature of their gene expression. This involves implementing stringent measures to control the false positive rate (FPR), thereby maintaining a high level of accuracy. By carefully eliminating false positives (FPs) and employing targeted RNA-seq panels, it is possible to achieve robust variant detection for genes that are expressed even in the absence of DNA-seq findings. These strategies collectively underscore the critical role of RNA-seq in both complementing DNA-seq and independently informing precision medicine.

In clinical diagnosis, drug development, and therapeutic decision making, understanding the impact of genetic variants on protein expression (“the target”) and function is crucial. For example, a DNA mutation in a gene that is never expressed in a certain cell type or tissue in question will have less consequence versus those expressed. This study aimed to thoroughly assess variant detection using targeted next-generation sequencing (NGS) data, offering a comprehensive guide for testing clinical oncopanel sequencing focusing on two common scenarios: (1) using RNA-seq results to verify and prioritize DNA variants; (2) Using RNA-seq independently to detect variants. We employed four targeted panels to detect variants in paired RNA and DNA NGS data with a reference sample created by our previous study, where a ground truth DNA variant set and a high-confidence negative position list were established^20^. The list of high-confidence negative positions is indispensable for calculating the FPR and its control by adjusting the parameters in bioinformatics pipelines. The variant detection results enabled a detailed comparison, highlighting the potential of RNA-seq to validate and augment DNA-seq findings. The analysis focused on ensuring high accuracy in variant detection and exploring the performance of RNA-seq across different panels and methodologies in different scenarios. By doing so, we aimed to evaluate approaches for further validating the detected DNA variants by comparing them to targeted RNA-seq expressed mutations, and to provide insights that enhance the precision and reliability through a more comprehensive somatic mutation detection strategy, which is pivotal for advancing precision medicine and improving patient outcomes.

## Results

**Fig. 1:**
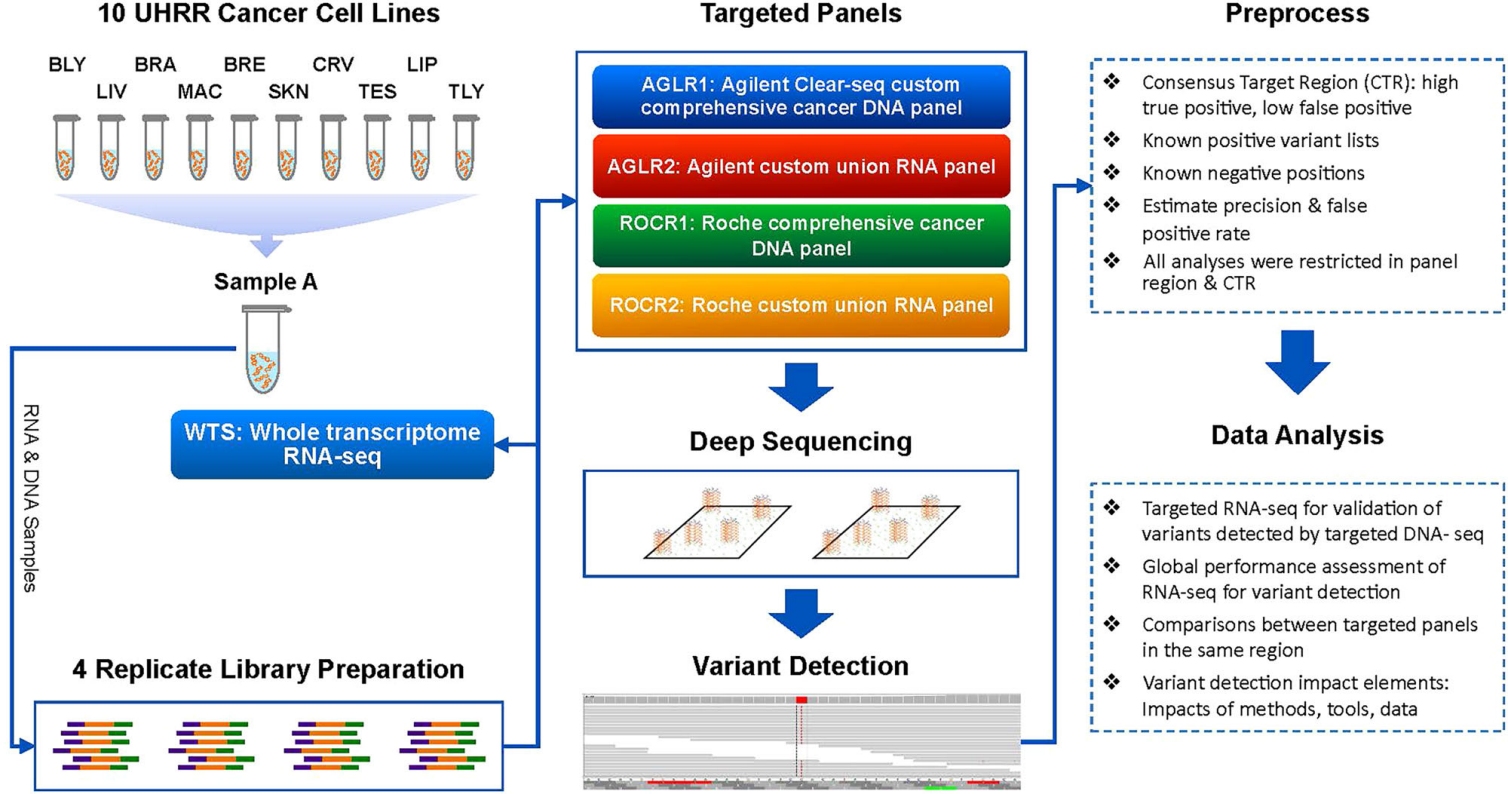
Study design. Matched RNA and DNA reference samples were extracted and sequenced using four targeted panels, with library replicates for comprehensive comparison of variant detection against the known variants characterized in the DNA reference sample. The DNA reference sample was constructed by an equal mass mixture of the DNA samples extracted from the ten cancer cell lines used to make the RNA reference sample, i.e., Agilent Universal Human RNA Reference (UHRR) sample. Although AGLR1 and ROCR1 were originally designed as DNA panels, RNA was also captured after the reverse transcription to cDNA and sequenced using these panels to assess their applicability for variant detection. AGLR2 and ROCR2 were specially designed for comprehensive analysis. These research panel designs incorporated targets from eight established onco-panels and included additional gene sets considered of interest to the community, forming the basis of these custom union panels. See our data descriptor paper for details^47^. In comparison, whole transcriptome RNA-seq (WTS) with poly(A) enrichment was also conducted for UHRR.

In this study, four targeted panels were used to assess variant detection in paired targeted DNA-seq and RNA-seq data generated for the reference samples (Fig. 1). These panels (the Agilent Clear-seq Custom Comprehensive Cancer DNA panels [“AGLR”] and the Roche Comprehensive Cancer DNA panels [“ROCR”]) included AGLR1 and ROCR1, which are DNA panels, and AGLR2 and ROCR2, which are RNA panels. These panels were designed with specific characteristics to target key genomic regions or transcripts of interest. The AGLR panels were designed with longer probes (120 bp), while the ROCR panels utilized shorter probes (~70–100 bp). Usually, RNA panels have some exon-exon junction covering probes to capture RNA-specific variants. DNA panels may have probes extending into the intron regions. Additional details about the panels’ designs, probe lengths, and targeted regions are provided in the Methods section. Additionally, whole transcriptome RNA-seq (WTS) data were included to evaluate the benefits and disadvantages of targeted RNA-seq approaches. Analysis was limited to the intersection of each panel’s targeted regions and the pre-defined high-confidence Consensus Target Region (CTR)^20,21^ (Methods). Known positive (KP) variants and known negative (KN) positions from the reference sample study^20^ served as the ground truth in this study to evaluate the performance and calculate the FPR.

### RNA-seq confirms and prioritizes clinically relevant DNA variants

**Fig. 2:**
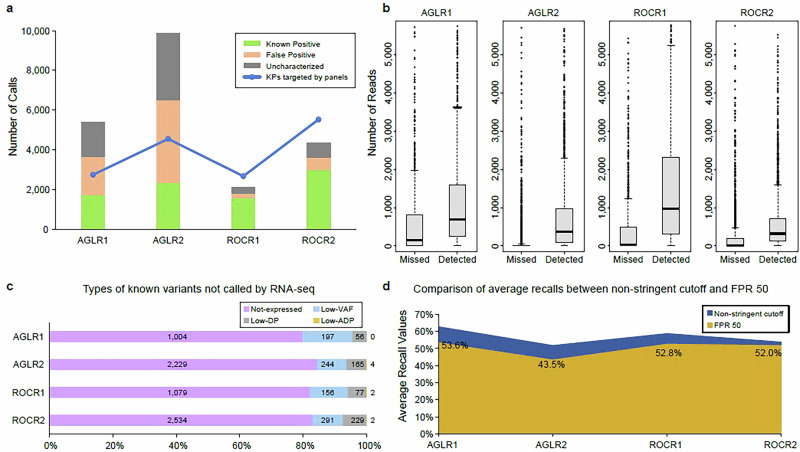
Using RNA-seq results to verify and prioritize DNA variants. **a** The numbers of different types of calls reported by various panels without controlling the FPR. The “true set” was established in our previous study using the same reference samples. Variants not included in the “true set” were categorized as uncharacterized calls. The blue line represents verified true variants that are targeted and sequenced by the specific panels (AGLR or ROCR), meaning they fall within the regions targeted by the probes in the panels. **b** Comparison of expression levels between two groups of known positive variants: those detected and those missed by targeted RNA-seq across different panels. The number of reads (Y axis) was set to zero if a call was absent in the expression results. A Wilcoxon Rank Sum Test was applied, resulting in a significant p-value of 2.2e-16 for all panels. **c** Known positive variants missed by targeted RNA-seq data. Not-expressed: not detectably expressed. For example, it may be fairly expressed but the bait performance is poor. Low-VAF: calls were expressed but had a VAF < 2%, Low-DP: expressed calls with VAF ≥ 2% but had a DP < 20, Low-ADP: expressed calls with VAF ≥ 2% and DP ≥ 20, but ADP < 2 (=1). **d** Comparison of average recall values across panels, considering conditions with non-stringent cutoff versus where the FPR was reduced to 50 per million bases.

To gain an overview of the capacity of targeted RNA-seq approaches to detect transcribed DNA variants, we aimed to recall as many variants as possible in each panel by including all variant calls from all three callers, including VarDict^22^, Mutect2^23^, and LoFreq^24^, adopted by an in-house assembled bioinformatic pipeline modified from SomaticSeq^25^ (Methods). To this end, a relatively conservative approach was adopted, where variants with a variant allele frequency (VAF) ≥ 2%, a total read depth (DP) ≥ 20, and an alternative allele depth (ADP) ≥ 2 were considered, minimizing explicit control of the false positive rate (FPR) for RNA-seq results (Methods). We then compared the targeted RNA-seq results with the true set identified from the reference samples in our previous study^20^ (Methods). With the indicated cutoffs, the Agilent Clear-seq Custom Comprehensive Cancer DNA panels (“AGLR1 and AGLR2”) reported a significant number of FPs and uncharacterized calls, which were defined as neither known positive (KPs) nor known negative (KNs) (Fig. 2a). In contrast, Roche Comprehensive Cancer DNA panels (“ROCR1 and ROCR2”) reported substantially fewer of these calls. Additionally, fewer calls were detected in the WTS results within the panel regions (Supplementary Fig. 1). This finding is consistent with the broader but shallower coverage provided by WTS compared to the deeper, more focused sequencing of these targeted panels. While the targeted panels offer enhanced detection capabilities for low-frequency variants, the non-stringent cutoffs compounded by current bioinformatics limitations can potentially lead to incorrect signal interpretation and an increased number of false-positive calls.

Notably, when compared to the total number of KPs targeted by the panels, only 51.3% (AGLR2) to 63.1% (AGLR1) were detected by targeted RNA-seq (Fig. 2a), even under a non-stringent cutoff. Although AGLR1 was designed as a DNA panel, RNA was also captured after the reverse transcription to double-strand cDNA and sequenced using this panel to assess its performance of variant detection through targeted RNA-seq. By investigating the coverage of the KP variants detected and missed by RNA-seq, significant differences were observed from all panels (Fig. 2b), indicating that the variants not detected by RNA-seq had low expression levels. Further detailed investigation of the missed KP variants revealed that over 80% of these variants were not expressed, hence not found in the RNA-seq results. The remaining variants were filtered out due to either low VAF, low DP, or low ADP (Fig. 2c). This finding suggests that RNA-seq can complement DNA-seq by highlighting variants that are transcribed and hence potentially functional, providing a more robust and comprehensive variant impact understanding.

The use of a conservative approach provided us with a landscape of the variants detected by targeted RNA-sequencing, but the excess of FP calls makes this approach unsuitable to conduct a fair comparison across panels. To this end, we implemented a more focused approach by fixing for all panels the estimated FPR to 50 FP calls per million bases, leveraging the “truth” set from the reference DNA samples (Methods). We first focused on the FP calls identified by their VAF, DP, ADP, and the individual callers who reported them. As a result, most of the FP calls were reported by VarDict alone and only had two ADPs (Supplementary Table 1). However, VarDict appeared more sensitive, and the FP calls it identified had significantly lower coverage than the KP variants (Supplementary Table 1). To enhance accuracy, we set a rule, different from the non-stringent technical cutoff, requiring a variant call to be reported by at least two out of three callers, and have an ADP greater than three. We then adjusted the VAF cutoff to achieve an FPR of approximately 50 per million bases for all the panels (Methods). We then compared the recall before and after the FPR control across panels (Fig. 2d). All panels missed some additional KP variants with such a cutoff. Compared to the AGLR panels, the ROCR panels missed fewer KP variants as they had fewer FP calls to be removed initially. Ultimately, 43.5% to 53.6% of the KP variants were confirmed by RNA-seq data, with the FPR controlled. This highlights that the selection of cutoffs significantly impacts the KP variants detection and validation.

A concordance assessment between DNA-seq and RNA-seq variant VAFs was conducted. We compared the VAFs of KP variants detected in RNA-seq to their corresponding values in the DNA-seq true set for each targeted panel, using Rep-1 as an example. Supplementary Fig. 2 presents scatter plots illustrating the relationship between true DNA-seq VAFs (*x*-axis) and RNA-seq detected VAFs (y-axis) for each panel. We observed a strong correlation between DNA-seq and RNA-seq VAFs across all panels, with R² values consistently around 0.85, indicating good agreement between the two sequencing approaches. While most variants followed a linear trend, some deviations were noted, which may be attributed to differences in gene expression levels, allele-specific expression, and RNA processing effects.

### Independent variant detection through RNA-seq identifies clinically actionable mutations

**Fig. 3:**
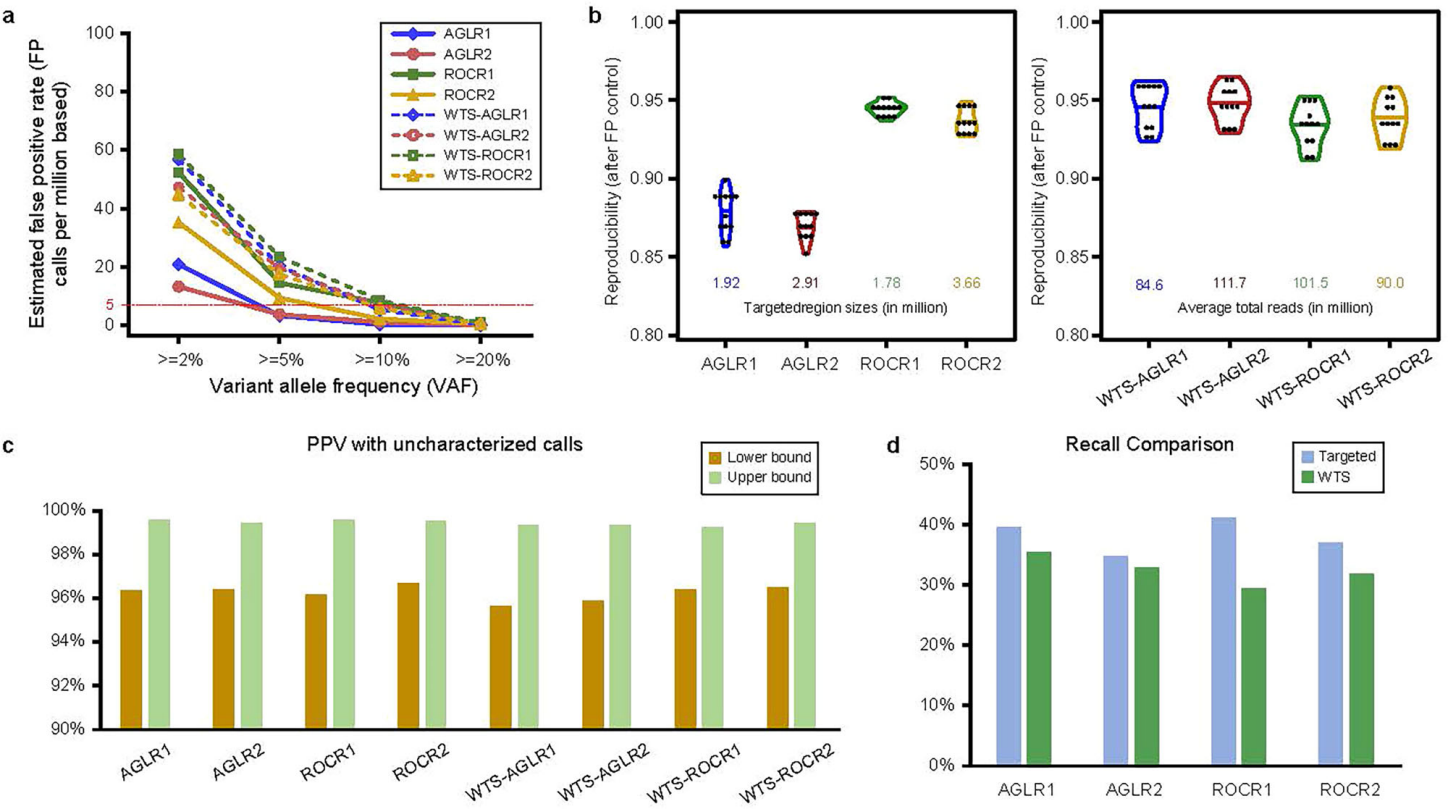
Independent variant detection using RNA-seq. **a** The estimated FPR for each targeted panel and WTS results, restricted to panel regions. Various VAF cutoffs were applied for each panel and data type to achieve an FPR of 5. **b** Reproducibility measurements of targeted RNA-seq and WTS results, restricted in panel regions, after the FPR to 5 per million bases. **c** PPV estimates by panel (average), including WTS results within each panel region. The upper bound PPV was calculated by considering all unknown variants as true positives. Conversely, the lower bound PPV was obtained by treating all unknown variants as negatives. This approach provides a range of possible precision values, accounting for the uncertainty in RNA-unique variant classification. **d** Comparison of recall rates for targeted RNA-seq and WTS results after controlling the FPR to 5 per million bases. Recall is defined as the proportion of known positive variants successfully detected by each panel, highlighting performance differences across sequencing methods.

Importantly, there are scenarios where RNA-seq may need to be used independently for variant detection, such as when DNA-seq data is unavailable or when investigating gene expression-specific variants. In these cases, the performance and reliability of RNA-seq alone become critical. We therefore explored the benefit of using RNA-seq as a standalone method for variant detection by assessing its potential to accurately identify variants. To ensure accurate variant detection with RNA-seq alone, it is essential to control the FPR rigorously by avoiding minimal or non-stringent cutoffs. Starting with the cutoff criteria set as: agreement between at least two out of three callers, VAF ≥ 2%, ADP ≥ 4, and DP ≥ 20, we first evaluated the estimated FPR of targeted panels and the WTS results in panel regions. As expected, most FP calls occurred at low VAF ranges (≤5%) for all panels (Fig. 3a). With these cutoffs, the estimated FPRs for different panels and data types ranged from 13 to 60 per million bases. The ROCR panels exhibited higher FPRs compared to the AGLR panels, indicating differences in panel designs and sensitivity to FPs. Additionally, more FP calls were found in the WTS data than targeted panels within the same regions.

We noticed that the FPR of ROCR1 was unexpectedly high within VAF range ≥5%. Further examination revealed that a cluster of 10 FP calls within a tiny region was the major contributor, resulting from incorrect variant calling by the pipeline (Supplementary Table 2). Most of these calls were specific to the ROCR1 panel. None were found in any DNA-seq results, and only two and four were identified in the AGLR2 and ROCR2 targeted RNA-seq panels, respectively. Notably, most of them were also detected in WTS data within the ROCR1 panel regions. Additionally, these variants were classified as “clustered events” by Mutect2, which is indicative of FP calls (Supplementary Table 2). Considering these FP calls were clustered, panel specific, had high VAF, and could be detected only under certain settings, we excluded these 10 calls from the rest of the analysis to strengthen the control of FPR for RNA-seq data. Furthermore, to ensure the accuracy in the utilization of RNA-seq independently for variant detection, the FPR must be reduced to a minimal level. To reach a FPR of 5 per million bases, we increased the VAF cutoff for each panel. Due to the differences across panels, the required VAF cutoffs on average varied: AGLR1 at 4.4%, AGLR2 at 3.7%, ROCR1 at 5.2%, and ROCR2 at 5.7% (Supplementary Table 3).

After the FPR was reduced to a minimal level, we then evaluated the overall variant detection performance of targeted RNA-seq and WTS data independently. Reproducibility was calculated across pairs of individual replicates, serving as an indicator of the stability of the panels. For targeted RNA-seq data, reproducibility rates ranged from 85% to 95%, with the AGLR panels showing lower values than the ROCR panels (Fig. 3b, Methods). However, values were similar within the same panel set, although the panel types and sizes varied. This might be due to inherent differences in design or technical variability between panels. Notably, WTS results exhibited higher and more stable reproducibility (~94% for all panel regions), likely because the WTS results originated from the same sequencing run, and the observed differences were only due to the specific regions analyzed (Fig. 3b).

To reduce the FPR for the WTS results to the minimal level, higher VAF cutoffs were required compared to targeted RNA-seq: AGLR1 at 9.3%, AGLR2 at 9.3%, ROCR1 at 9.5%, and ROCR2 at 9.0% (Supplementary Table 4). We then evaluated the positive predictive values (PPVs), calculated as the ratio of known positive variants to the total variants called by each panel or in panel regions by WTS. Given the presence of uncharacterized calls reported by RNA-seq, we calculated both upper (about 99.5%) and lower (about 96%) bounds of PPVs for an average of the four replicates, classifying all the uncharacterized calls as either positives or negatives, respectively (Methods). As we have reduced the FPR for all the panels and WTS results in panel regions to the same minimal level, similar and high PPVs were observed for all the datasets (Fig. 3c). The findings suggested that stringent control of FPR yielded high PPVs across different sequencing approaches. Even though we observed similar levels of PPV across all datasets, some differences in recall were noted. Targeted RNA-seq consistently showed higher recall values than WTS (Fig. 3d). This highlights the benefit of targeted RNA-seq for variant detection, as it provides deeper coverage in specific regions, allowing for the detection of more positive variants.

### Comparative analysis of variants identified in overlapping panel regions

Different panels demonstrated varying performance in terms of reproducibility and FPR when analyzed individually (Fig. 3). To understand the varying performance in more detail, we sought out to evaluate the consistency and accuracy of variant detection across the different panels within their overlapping target regions. To achieve this, we conducted a detailed comparison focusing on panels from the same vendor (AGLR1 and AGLR2) and the same panel type (AGLR2 and ROCR2, RNA panel).

**Fig. 4:**
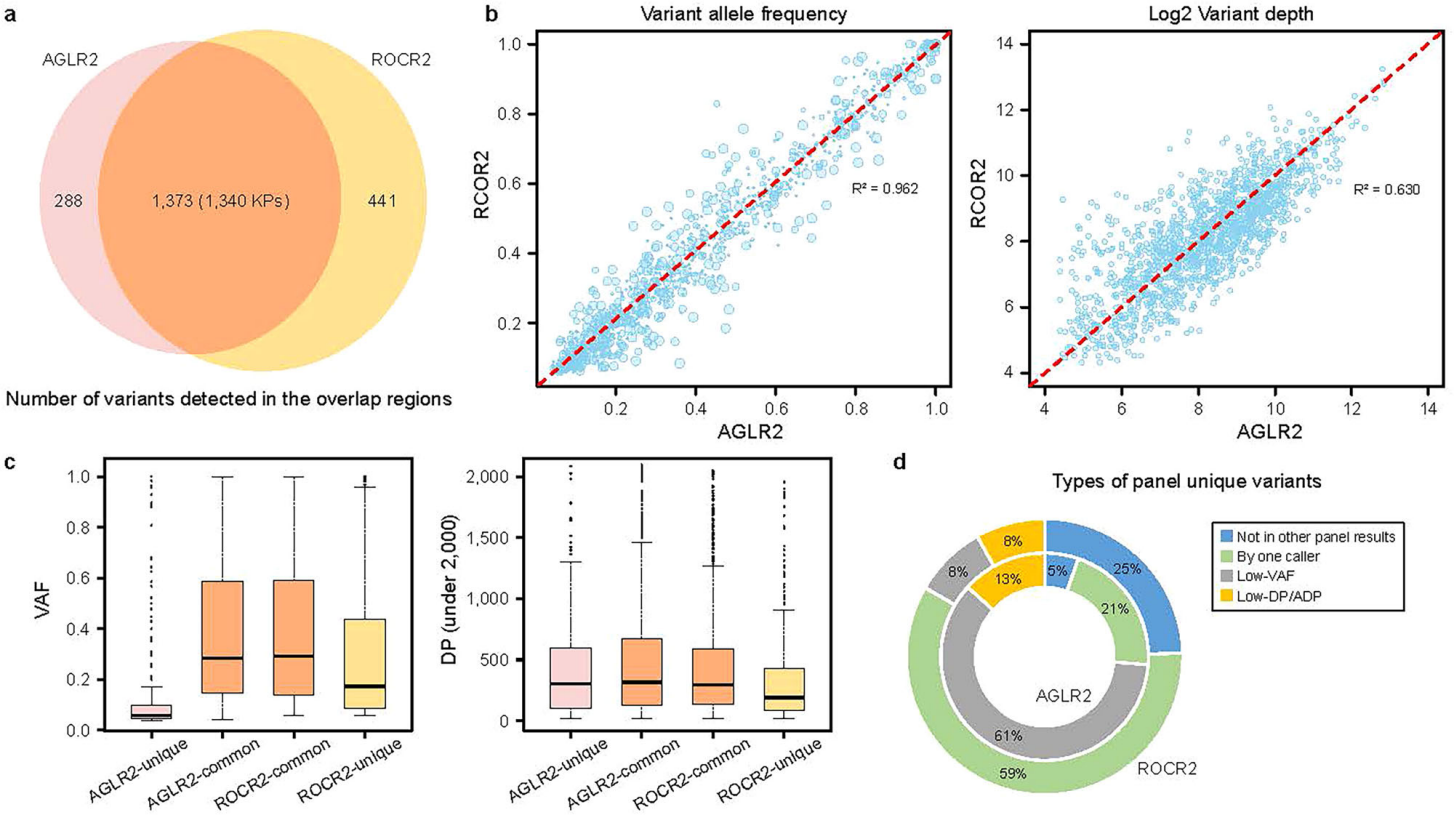
Comparison analysis of variant detection between AGLR2 and ROCR2 in the overlapping region (replicate 1 as an example). **a** The number of variants detected in the overlapping region by AGLR2 and ROCR2 excluding known FP calls. **b** Comparison of the log2 variant depth (larger symbol sizes indicate lower variant position depths) and VAF for common variants shared between AGLR2 and ROCR2 in the overlapping region. **c** Distributions of total DP and VAF per variant category, comparing panel-unique and common variants to both. **d** Percentage of the four types of panel-unique variants explaining why the other panel missed them.

The overlapping regions between AGLR1 and AGLR2 encompassed 1.49 million bases. For AGLR2 and ROCR2, the overlapping regions included 2.89 million bases. Using one of the four replicates as an example, we observed the variant call concordance scores of 85.2% and 86.1% in the overlapping regions of AGLR1 and AGLR2, respectively (Supplementary Fig. 3). Notably, the reproducibility based on replicate pairs was approximately the same in AGLR panels (Fig. 3b), indicating consistency in the results of these two panels from the same vendor. The differences observed in the overlapping regions of AGLR1 and AGLR2 can be partly attributed to the boundary effect caused by the cutoff applied^21^. As shown in Fig. 4a, 82.7% of AGLR2 variant calls in the overlapping regions were also reported by ROCR2, with a reciprocal confirmation rate of 75.7% for ROCR2 calls, resulting in 288 and 441 panel-unique variants from each side, respectively.

Overall, we observed high consistency in reported variant position depth and VAF between the two panels for common variants (Fig. 4b). The *R*^2^ for VAF was notably high at 0.962, significantly surpassing the *R*^2^ of 0.630 for log2(DP). This high *R*^2^ value for VAF supports the accuracy of RNA-seq in detecting genetic variants and highlights the intrinsic properties of the genetic variants. Variants with larger differences in VAF between the two panels were associated with low variant position depths, as indicated by larger dot sizes, with the smallest DP = 20 (Fig. 4b). The considerable variability in variant depth might impact the variant calling pipeline and drive differences in the results. For panel-unique variants, we observed a similar distribution of variant depth compared to shared variants. However, the VAFs of these unique variants were significantly lower, particularly for AGLR2-unique variants (Fig. 4c). Additionally, a boundary effect at the VAF cutoff was observed, which could also contribute to the detection of panel-unique variant calls.

Further investigation revealed various possible reasons driving panel-unique variants. Specifically, 271 AGLR2-unique variants found in the ROCR2 results were filtered out due to either being reported by only one caller or having lower than cutoff VAF, DP, or ADP. To achieve the low FPR, different VAF cutoffs were required for AGLR2 and ROCR2, set at 3.7% and 5.7%, respectively. The largest portion (61%) of AGLR2-unique variants were those detected in AGLR2 but failed to pass the higher VAF cutoff for ROCR2. In contrast, the majority (59%) of ROCR2-unique variants were initially detected by only one caller in the AGLR2 results and subsequently filtered out. We also found that 25% of the ROCR2-unique variants were detected by no caller in the AGLR2 data (Fig. 4d). The detection of panel-unique variants highlights the impact of panel-specific cutoffs and the inherent variability in bioinformatics pipelines. These discrepancies suggest that while some panels may excel in sensitivity, others offer greater specificity, necessitating a balanced approach in panel selection and variant calling based on the clinical or research context.

### Impact of bioinformatics factors on variant detection

**Fig. 5:**
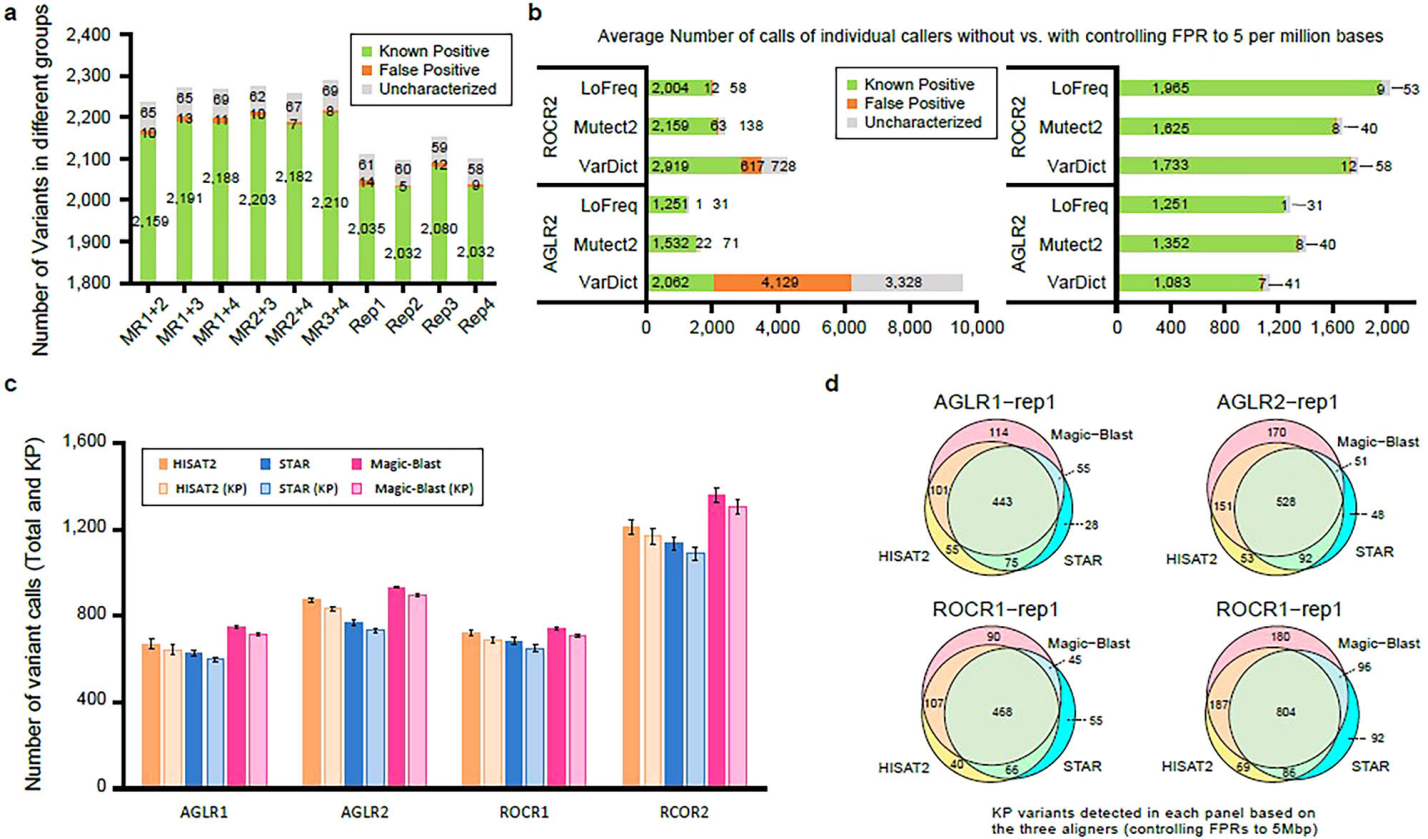
Impact of bioinformatics factors on variant detection. **a** The number of variants detected in each individual library, including known positive, false positive, and uncharacterized calls, comparing single-library and merged-library approaches, with ROCR2 as an example. MR1 + 2 represents the library prepared by merging replicates 1 and 2 of ROCR2. **b** Comparison of the average number of calls detected by individual callers used in this study in AGLR2 and ROCR2 without and with controlling the FPR to 5 per million bases. **c** The average numbers of total and known positive calls detected by pipelines based on different aligners in each panel. The error bars represent the variability across four replicated libraries, calculated as the standard deviation of the data. **d** The number of variants (excluding FP calls) detected by different pipelines after controlling the FPR to 5 per million bases. An impressive number of variants were found to be aligner-specific or shared by two aligners across all panels.

To further investigate the impact of increased coverage on variant calling efficacy, we generated a merged-library dataset by combining the alignment files of two replicates for the ROCR2 panel. Six merged-library samples were created (replicates 1&2, 1&3, 1&4, 2&3, 2&4, 3&4) and used for calling variants using the same pipeline. The median variant depth of the merged-library samples (~600) was about twice that of the single-library samples (Supplementary Fig. 4). We next compared the number of variants detected in single and merged libraries after controlling for the FPR. As expected, we observed similar numbers of FP calls on average (10 vs. 10). However, with higher coverage in merged libraries, more true positives on average (2189 vs. 2045) were detected. Taken together, these results demonstrate the benefits of higher coverage for improving the sensitivity of variant detection (Fig. 5a).

The bioinformatic methods employed, such as read aligners and variant callers, significantly impacted the results of variant detection. We required the agreement of two callers to make a call when using RNA-seq alone for variant detection to boost confidence. Here, we compared the different types of calls made by individual callers. Without controlling the FPR, VarDict detected the highest number of KP variants in AGLR2 and ROCR2 (Fig. 5b). However, it also reported a much higher number of FP and uncharacterized calls compared to the other two callers. Mutect2 detected more KP calls while yielding a comparable number of FP and uncharacterized calls compared to LoFreq. Then, we elevated the VAF cutoff for each caller to control the FPR. Setting the FPR to 5 per million bases resulted in the loss of some KP variants. Notably, using these criteria, VarDict reported the lowest number of KP variants, as over 800 were filtered out. (Fig. 5b).

We further investigated the impact of different RNA-seq mapping methods by employing three commonly used aligners for RNA-seq data: HISAT2^26^, STAR^27^, and Magic-Blast^28^. Mutect2 was chosen due to its compatibility with many aligners and its ability to detect the highest number of KP variants while controlling the FPR to 5 per million bases (Fig. 5b). To ensure high-confidence results, VAF cutoffs were set for individual aligners to achieve the FPR of 5 per million bases (Supplementary Fig. 5). As a result, the Magic-Blast-based pipeline yielded the largest number of total calls and KPs, followed by HISAT2, with STAR reporting the fewest (Fig. 5c). The pattern was consistent across all panels and library replicates. When controlling the FPR to 5 per million bases, these pipelines, consisting of a single caller Mutect2, reported significantly fewer total calls and KP variants compared to the assembled method. A large portion of the variants were commonly detected across different aligners, resulting in a small yet considerate portion of aligner-unique variants (Fig. 5d). There was strong agreement (*R* > 0.95) in VAF and DP of common variants across the various aligners (Supplementary Fig. 6A). However, differences in VAF were observed among the aligner-unique variants. STAR-unique variants showed lower VAF values than those detected based on other aligners (Supplementary Fig. 6B).

Another evaluation on the impact of VAF and alternate (ALT) DP thresholds on RNA-seq variant detection was conducted based on the three aligners. We assessed multiple filter combinations by measuring the average number of total variant calls, KP calls, and FP calls across replicates (Supplementary Fig. 7). The results demonstrate a consistent pattern across all aligners. Higher VAF and ALT read thresholds led to a reduction in total variant calls, while effectively lowering FPR and improving precision. Notably, KP calls remained relatively stable, suggesting that parameter adjustments primarily refine variant calls without significantly compromising sensitivity. Conversely, lower thresholds resulted in a sharp increase in FPs, highlighting the importance of careful parameter selection. These findings confirm that fine-tuning filtering criteria is essential for optimizing RNA-seq variant calling while maintaining a balance between sensitivity and specificity.

### Clinical significance of RNA-unique variants in enhancing precision oncology

Lastly, we explored the relevant and potential impacts of RNA-unique variants on human diseases. RNA-unique variants can provide critical insights into gene expression dynamics and their potential role in disease, especially when these variants influence transcript splicing, regulation, or protein function. To achieve a direct and fair comparison, we used targeted DNA-seq data for each panel to detect variants and compared it against paired RNA-seq results. For DNA-seq data, we applied cutoffs of VAF ≥ 2%, ADP ≥ 4, DP ≥ 20, and required agreement from at least two out of three callers to ensure a low FPR. As a result, at most one FP call was reported in any replicate, implying FPR below 1 per million bases. For RNA-seq data, we maintained the FPR at 5 per million bases and excluded known FP calls from the comparison. We identified variants only present in the RNA-seq results for each replicate, collecting a union set for each panel. This resulted in 201 RNA-unique variants (147 exclusive variants): AGLR1 with 30, AGLR2 with 79, ROCR1 with 44, and ROCR2 with 48 (Supplementary Table 5). All these variants met the established cutoffs for each panel and had high VAF and variant DP, indicating the reliability of these calls (Supplementary Fig. 8). Taking the AGLR2 panel as an example, we found that 29 out of 79 RNA-unique variants were absent in all the DNA replicates; 35 of the remaining 50 were only detected by one caller (VarDict), and the rest (15) were filtered out due to VAF (5) or DP (10). These results suggest that while these RNA-unique variants tended to be real calls, a fraction of these may be missed by DNA-seq due to low signal.

We employed SnpEff and SnpSift^29^ to identify the relevance and putative impacts on human disease of the 147 RNA-exclusive variants across all four panels based using ClinVar data. These 147 variants were observed after excluding duplicates from different panels. As a result, 90 variants were predicted to have either HIGH or MODERATE impacts (Supplementary Table 5). The affected feature type, based on Sequence Ontology (SO) terms, included frameshift_variant, missense_variant, missense_variant & splice_region_variant, protein-protein_contact, sequence_feature, stop_gained, structural_interaction_variant. Additionally, 33 RNA-unique variants were annotated with ClinVar clinical disease name (CLNDN) such as: non-small cell lung cancer, familial cancer of breast. Notably, 20 out of these 33 annotated variants were predicted to have HIGH or MODERATE impacts. To further strengthen the interpretation of these RNA-unique variants, we cross-referenced them with the Catalog of Somatic Mutations in Cancer (COSMIC) database^30^ to determine if any had been previously reported in cancer. COSMIC is a cancer-specific mutation database, and identifying matches provides additional evidence supporting the relevance of these variants in oncogenesis. We found that 29 out of the 147 RNA-unique variants were also recorded in the COSMIC database. Among these, 16 variants were identified to have HIGH or MODERATE impacts by SnpEff and SnpSift. The COSMIC cross-referenced data have been included in Supplementary Fig. 5. These results indicate that the RNA-specific variants, after stringent FP control, are likely to be real and have potential impacts on human diseases such as cancers. This analysis also demonstrates the potential utility of RNA-seq in confirming and prioritizing actionable variants, highlighting its value in clinical and research settings.

### Impact of probe and exon length: no significant difference observed

The probe lengths of the two panels differed by design, with AGLR2 using 120 bp probes and ROCR2 using probes of 70–100 bp. We investigated whether these different probe lengths might cause inconsistent variant calling results. We reasoned that, in the case of short exons, longer probes would cover exon junctions, thus potentially decreasing the capture efficiency. We compared the expression of the exons using the TPMCalculator^31^ within the intersection regions of AGLR2 and ROCR2 panels. Although the panel with shorter probes (ROCR2) tended to detect higher exon expression, no major difference in capture efficiency due to probe length was observed (*R*^2^ 0.85, Supplementary Fig. 9). We then grouped the exons covering variants based on their lengths (Supplementary Fig. 10A). As expected, longer exons covered more variants, but with similar percentages across panels. The AGLR2 panel showed a slight difference, detecting fewer variants (in percentage) in smaller exons (≤200 bps). Supplementary Fig. 10B shows the percentage of KP variants per exon length. Longer exons (>120) covered more non-KP variants in ROCR2 (probe length of approximately 75–100) and WTS panels than in AGLR panels, having a 120 bp probe length. Despite the differences in probe lengths, there was no significant impact on capture efficiency or variant detection across the panels. Our results suggest that probe length variations do not substantially affect the performance of targeted RNA-seq panels in terms of detecting and capturing exonic variants.

## Discussion

Our findings revealed that RNA-seq confirmed about 50% of the KP variants detected previously by targeted DNA-seq across each of the four targeted panels. The variants missed by RNA-seq were typically characterized by low expression or weak signals, such as low VAF or insufficient coverage, consistent with previous studies^6,32^. Notably, many of the DNA-seq variants scored as unexpressed in this study were classified by COSMIC or other databases as relevant to disease or cancer^30^. Therefore, variants detected by DNA-seq but found not to be expressed at significant levels may require more sequencing or further validations to confirm their therapeutic and clinical relevance and to be implemented in practice. The results underscore the importance of integrating RNA-seq with DNA-seq to provide a more comprehensive assessment of variant significance and to prioritize variants with confirmed expression for clinical decision making. A potential strategy to enhance detection concordance between RNA-seq and DNA-seq involves deeper or more extensive sequencing, which could capture low-expressed variants (e.g., transcription factors) more effectively. With the recent advancements in sequencing technologies, such as the Illumina NovaSeq X-Plus system, the associated costs of sequencing are expected to drop by 40–80%, making this approach more feasible. Considering that cancer therapeutics costs can exceed $25 K per month, improving the accuracy of therapeutic assignments through enhanced multimodal (matched DNA and RNA) sequencing, which is of a relatively low cost versus a cancer therapeutic, could significantly aid both financial efficiency and clinical outcomes.

RNA-seq demonstrated strong efficacy in variant detection, standing out even when used independently. Despite the inherent differences in panel design, the results consistently showed high reproducibility, precision, and an acceptable FPR. Notably, targeted RNA-seq panels matched the performance of WTS in overlapping regions, offering superior recall and significantly lower FPR, particularly for low VAF variants (≤5%). Crucially, RNA-seq uncovered novel, clinically significant variants missed by DNA-seq, highlighting its critical role in accurate variant detection. These findings reinforce that targeted RNA-seq is not only a reliable tool for identifying clinically relevant mutations but also an essential complement to DNA-seq, advancing precision medicine by validating and prioritizing key variants. The discovery of RNA-unique variants with high predicted impact further underscores the potential of RNA-seq to reveal biomarkers or therapeutic targets that may be overlooked by DNA-seq alone, with profound implications for the development of targeted therapies, especially for cancers driven more by changes in gene expression than by DNA mutations.

Achieving a high level of accuracy often comes with trade-offs. To achieve a lower FPR, some true positives were missed due to the more stringent cutoff that was applied. In this study, when reducing the FPR from 50 to 5 for targeted RNA-seq data, the recall decreased by 9%–15%, depending on the panel. This trade-off underscores the balance between minimizing FPs and maintaining a high recall rate (sensitivity) in variant detection. To ensure high accuracy and reliability of the results, a conservative FPR is recommended in a scenario of using RNA-seq alone for variant detection. In this study, it was demonstrated that when rigorously controlled for FPs, RNA-seq can serve as an effective standalone method for variant detection. This capability is particularly valuable in scenarios where DNA-seq data is unavailable or where RNA-specific insights are essential, thus broadening the scope and utility of RNA-seq in precision medicine.

The VAF is a critical threshold for variant detection, particularly in cancer, where the presence of non-tumor cells dilutes the VAF of tumor-specific variants. Variant detection accuracy can be significantly impacted by the statistical models used by variant calling algorithms and hard VAF cutoffs. In our study, we integrated three variant callers and adopted a consensus approach that requires agreement from at least two callers, as described in the Methods section. This consensus approach effectively mitigated the impact of low VAFs on variant detection. However, the boundary effect of the VAF cutoff still played a role in some cases, especially when comparing variants identified across different replicated libraries or methods. For example, our findings demonstrated that some panel-unique variants with low VAF passed the cutoff in one panel but failed in another, highlighting the challenges and considerations needed when setting and interpreting VAF thresholds. This boundary effect underscores the importance of careful VAF threshold selection and the potential need for more flexible or adaptive approaches to improve variant detection consistency across different datasets.

Differences in coverage depth and target specificity, which directly impact the accuracy of variant detection, are likely contributors to the lower accuracy of WTS. While targeted RNA-seq offers superior specificity within its focused regions, WTS provides a broader, discovery-based approach with a more comprehensive view of the transcriptome, although at the cost of higher FPR. The varying performance of different bioinformatics tools highlights the need for careful selection and optimization to ensure accuracy in RNA-seq analysis. Proper cutoffs for VAF and DP, alongside strategies such as majority voting involving multiple tools, are essential for minimizing errors. Failure to implement these measures can lead to misinterpretations in clinical practice, potentially impacting diagnostic decisions and treatment strategies. Moreover, the variability observed across different bioinformatics tools and settings underscores the critical importance of pipeline optimization tailored to specific applications. As precision medicine advances, refining these pipelines to balance sensitivity, specificity, and reproducibility will be key to ensuring the reliability of RNA-seq as both a clinical diagnostic and research tool.

Importantly, both expressed and non-expressed genetic variants may be actionable. For instance, oncogenic, gain-of-function mutations are clinically relevant if they are expressed and have a functional consequence. Oncogenic mutations of *EGFR* gene are frequent in non-small cell lung cancer, and *KIT* or *PDGFRA* activating alterations are a hallmark of gastrointestinal stromal tumors. These mutations determine sensitivity/resistance to tyrosine kinase inhibitors^33,34^. Similarly, *KRAS* mutations are very common in many solid tumors, and targeted small molecule therapies, including Krazati® (adagrasib) and Lumakras® (sotorasib), have been approved for *KRAS*-G12C mutations. Numerous other targeted therapies addressing various *KRAS* mutations are in clinical development^35–38^. Importantly, the expression of the mutant gene may be lost during tumor progression as a mechanism of escape to drug inhibition^39^. In these cases, the sole evaluation of genomic DNA may erroneously indicate potential drug sensitivity while the target could be nonexistent (no longer expressed). Based on the results of this study, patients being considered for the targeted therapies could benefit significantly from additional RNA mutation profiling. In this case, RNA-seq can confirm the expression of the intended target in the tumor, ensuring that the mutations targeted by these therapies are not only present but also actively expressed. Conversely, loss-of-function mutations, such as nonsense or frameshift mutations in tumor suppressor genes, often undergo nonsense-mediated decay, reducing their detectability by RNA-seq but retaining significant clinical relevance due to their negative impact on protein function^40,41^. For example, with BRCA1/2 loss-of-function mutations that impair BRCA-related protein functions, determining tumor sensitivity to poly [ADP-ribose] polymerase (PARP) inhibitors is critical^42^. Therefore, RNA variant analysis has an important role. It is of utmost importance to distinguish between the application of RNA variants analysis in case of oncogenic/gain of function versus nonsense/frameshift loss-of-function mutations, especially in the case of low- or non-expressed variants. Acknowledging and accounting for non-expressed but actionable variants ensures that personalized treatment strategies are informed by a complete genetic landscape, enhancing the effectiveness of precision medicine.

One key observation in our study is that only 43–53% of variants detected in DNA-seq were also recovered in RNA-seq. This discrepancy is primarily due to the dynamic nature of gene expression, as not all mutations are transcribed at the time of sampling. However, it is important to consider several key aspects when interpreting these results. First, our study was conducted using cancer cell line models, which provide a controlled system for benchmarking RNA-seq variant detection performance. While these models are valuable for technical validation, they do not fully capture the biological complexity of tumor evolution in a patient. In clinical settings, tumor cells experience dynamic changes, and gene expressions may change alongside the tumor development. Second, the concern that a variant not expressed at diagnosis could later become a driver is a valid biological phenomenon. However, addressing this question would require molecular profiling in clinical studies, where gene expression changes are tracked over time, rather than relying on a single time point. Our study does not aim to predict future tumor evolution but instead focuses on the technical evaluation of targeted RNA-seq variant detection and its integration with DNA-seq analysis. Finally, before RNA-seq can be fully integrated into precision oncology workflows, its reliability, best practices, and performance metrics must be well-evaluated. Our study provides a technical foundation for future research and clinical studies to evaluate how RNA-seq can be effectively integrated into patient monitoring and treatment selection.

The identification and prioritization of neoantigens, essential for developing personalized cancer vaccines, can be significantly enhanced by integrating RNA-seq with DNA-based sequencing methods like WES^43^. NGS data is commonly used to map tumor-specific mutations by comparing the genetic profiles of tumor and normal tissues to establish if the mutation is of a somatic origin. However, the addition of RNA-seq provides critical insights into whether these mutations are actively expressed, a key factor in determining their relevance as potential neoantigens^44^. In addition, RNA-seq allows for the detection of alternative splicing events, frameshift mutations, and atypical splicing patterns, all of which may give rise to neoantigenic peptides that DNA-based methods might miss^45^. This ability to refine neoantigen selection is particularly valuable in the context of cancer vaccines, where only the most immunogenic and tumor-specific targets are desired. Targeted RNA-seq enhances the selection process by verifying the expression levels of these variants, allowing researchers to prioritize neoantigens that are most likely to be presented by tumor cells and elicit an immune response. RNA-seq also helps uncover novel splicing events and RNA-level alterations, expanding the range of potential neoantigens. Many companies are now leveraging targeted RNA-seq in cancer vaccine development to address these challenges. For example, therapies like the mRNA-4157 (V940) neoantigen vaccine rely on RNA-seq data to refine the selection of immunogenic targets, demonstrating the critical role RNA-seq plays in advancing personalized cancer treatments. This approach becomes a critical step in ensuring that the selected neoantigens are both expressed and biologically relevant, improving the overall efficacy of personalized cancer immunotherapies.

In this study, we analyzed SNVs and small indels ≤5 bases together, as many widely used variant callers report them in a unified format. In contrast, detecting moderate or large indels remains challenging^46^, and we lack sufficient data to thoroughly evaluate their detection performance and impact. Under minimized FPR control (VAF ≥ 2%, total DP ≥ 20, and ADP ≥ 2), small indels accounted for <5% of all detected variants. Among these indels, fewer than 10% were known positive variants, representing only 0.3% to 0.45% of all known variants. Furthermore, most indels that were not identified as known variants were detected by only one caller and exhibited low ADP, suggesting that these calls are likely low-confidence variants. These findings indicate that small indels had a minimal impact on the overall results, and their presence did not substantially affect our conclusions regarding targeted RNA-seq variant detection. Although small indels had a limited impact in this study, a more comprehensive investigation of indels, particularly larger ones, is needed. Future studies should explore the effects of indel size, sequencing biases, and probe design constraints to further refine RNA-seq variant detection strategies.

While answering many questions, this study does open the door for future work to explore the role of RNA-seq to further boost precision medicine. As mentioned earlier, the samples used in this study were, out of necessity, derived from cell lines and will not reflect the complex mixture of cell types present in a typical tumor. Thus, future work should include true clinical tumor specimens, with the consequence of not being able to know the ground truth. Moreover, this study does not seek to compare or recommend a specific panel provider over another. The choice of a specific panel will depend on the research and clinical question, type of tumor, variants of interest, specimen type, budget, and more.

## Methods

### Targeted panel design and sample preparation

The “Sample A” used in this study is an artificial sample previously developed and described with high variant density, generated by pooling 10 Agilent Universal Human Reference RNA (UHRR) cancer cell lines^20^. This reference sample was extensively analyzed, with variants and non-variant nucleotides mapped in a high-confidence consensus target region (CTR)^21^. In this study, DNA and RNA derived from Sample A (ten cancer cell lines) were used to prepare four replicate libraries. These libraries were sequenced using four targeted panels: AGLR1, AGLR2, ROCR1, and ROCR2, each designed and provided by the vendors (Fig. 1) to capture specific genomic regions or transcripts. Although AGLR1 and ROCR1 were originally designed as DNA panels, RNA was also captured after the reverse transcription to double-strand cDNA and sequenced using these panels to assess their applicability for RNA-based variant detection. In contrast, AGLR2 and ROCR2 were specifically designed to provide comprehensive RNA analysis. Alignments were conducted against the GRCh38.d1.vd1 reference genome. Both DNA-seq and RNA-seq adopted paired-end sequencing. The median read length for DNA-seq was 151 bp, while for RNA-seq, it was 100 bp. Due to variations in total reads and panel sizes, the estimated sequencing depth for DNA-seq varied widely, ranging from approximately 2,000X to 7,000X. In contrast, RNA-seq mapping rates were high and consistent, ranging from 94.5% to 97.5%, indicating robust alignment quality across samples (Supplementary Table 6). For a comprehensive breakdown of sequencing coverage, panel designs, sample preparations, SNP filtering criteria, and a complete list of detected somatic variants with their corresponding VAFs, please refer to our data descriptor paper^47^.

There are some notable differences between AGLR and ROCR panels. Firstly, the probe length of the AGLR panels was 120 bps, which is longer than ROCR’s average length of 75 bps (min 50 bps; max 100 bps). Additionally, while AGLR2 and ROCR2 panels were designed for almost the same regions of the genome, Agilent minimized the presence of exon-exon spanning probes. As a result, over 85% of the probes were fully contained within a single exon, making them suitable for DNA-seq as well. On the other hand, when Roche selected the probe candidates, individual exons were generally covered once in the exemplar transcript, and the set of exon-exon junctions was covered for every unique combination. For more details, please refer to our data descriptor paper^47^. These panels targeted different gene sets, with query regions covering 1.92, 2.91, 1.78, and 3.66 million base pairs within the CTR, and corresponding KP variants of 2741, 4551, 2669, and 5531 for AGLR1, AGLR2, ROCR1, and ROCR2, respectively. For further comparison, whole transcriptome sequencing (WTS) was performed to cover the entire CTR (21.65 million bps), including 37,668 KP variants. Small genetic variants were detected from DNA-seq and RNA-seq data using an in-house assembled bioinformatic pipeline modified from SomaticSeq^25^. The details of known variants detected (VAF ≥ 2%, total DP ≥ 20, and ADP ≥ 2) in RNA-seq data from four panels are listed in the Supplementary Table 7.

### Assembled variant calling pipeline—somaticSeq

To ensure robust and high-confidence variant detection, we utilized the SomaticSeq pipeline, an assembled framework that integrates multiple best-practice variant callers for somatic mutation detection. RNA-seq reads were aligned to the GRCh38.d1.vd1.fa reference genome using HISAT2 (v2.1.0), while DNA-seq reads were aligned using BWA-MEM (v0.7.15) to maintain consistency in genomic coordinate mapping. Post-alignment processing steps, including duplicate marking, splitting CIGAR strings, and base quality score recalibration (BQSR), were performed following GATK best practices. For variant calling, we integrated three widely used tools, MuTect2 (v4.0.5.2)^23^, VarDict (v1.5.1)^22^, and LoFreq (v2)^24^, within the SomaticSeq pipeline. Each tool was run with its default filtering settings, and SomaticSeq was used to integrate, filter, and refine the final variant set, leveraging its ensemble approach to enhance the results.

### Assessment based on known positive variants and known negative positions

Sample A was thoroughly analyzed in our previous studies^20^, where over 40,000 KP variants and 10 million KN positions were identified in the CTR. This region allows for higher accuracy and lower FPR than other genomic regions. This study restricted all panel results to the CTR to obtain high-confidence variants for further assessment and comparison. We leveraged KPs and KNs as a truth set to calculate the positive predictive value (PPV) and false positive rate (FPR).

### Calculation of variant expression

Read counts of the exons containing the variants were used to represent variant expression. HTSeq-Count^48^ was adopted to calculate the read counts. We created the feature file (GFF) by collecting the smallest exons that cover the target variants, and then calculated the expression of these exons.

### Calculation of reproducibility

Four replicate libraries prepared for each panel were used to calculate the reproducibility. The reproducibility between any two of the replicate libraries, designated as “LibA” and “LibB” was calculated as the portions of common variant calls between them. It is important to note that these reproducibility values are not symmetrical, as the total number of variants called in each library may vary. For each pair of replicates, there were in total 12 reproducibility values for each panel. We observed a boundary effect induced by the hard VAF cutoff across multiple replicates. For instance, the VAF cutoff for the AGLR2 panel was set at 3.7%. If a variant’s VAF was 3.8% in LibA but 3.6% in LibB, it should still be considered reproducible despite not being included in the final results of LibB. To account for this boundary effect, when determining the reproducibility of LibA to LibB, we applied the minimal VAF cutoff of 2% for LibB to reduce the impact of the effect. This approach ensured a more accurate representation of reproducibility between the libraries.

### Calculation of PPV lower and upper bounds

The PPV was calculated as the ratio of known positive variants to the total number of variants called by each panel or within panel regions by WTS. Since our truth set was incomplete, some calls were classified as unknown, meaning they are neither KPs nor KNs. Therefore, we calculated the upper and lower bounds of PPV for both targeted RNA-seq panels and WTS data. The true PPVs are expected to line within the range of lower and upper bound values observed.

1 $${Upper\; bound\; PPV}=\frac{{Number\; of\; KPs\; detected}+{Number\; of\; unknown\; calls\; detected}}{{Total\; number\; of\; calls\; detected}}$$

2 $${Lower\; bound\; PPV}=\frac{{Number\; of\; KPs\; detected}}{{Total\; number\; of\; calls\; detected}}$$

### Estimation of FPR based on known negative variant positions

In Sample A, over 10 million high-confidence KN variant positions in the CTR were pre-identified. Any call made at a negative variant position was considered a FP. The CTR is ~21.7 million bases, indicating that about half of these positions are unknown. Therefore, our FPR estimation might be conservative, as some calls remain uncharacterized, being neither conclusively KP nor KN. We estimated the FPR in each panel and CTR as the number of FP calls per million bases:

3 $${Estimated\; FP\; rate}=\frac{{Number\; of\; variants\; detected\; at\; KN\; positions}\,}{{Known\; negative\; size\; in\; panel\; \& \; CTR}}* 1,000,000$$

### Aligner comparison pipeline

To assess the impact of different aligners on variant detection, we performed a systematic evaluation using three RNA-seq aligners: HISAT2 (v2.1.0), STAR (v2.7.8a), and MagicBlast (v1.5.0). This comparison aimed to determine how differences in alignment strategies influence variant calling outcomes (Supplementary Fig. 5). Raw RNA-seq reads from the AGLR1, AGLR2, ROCR1, and ROCR2 panels were aligned to the GRCh38.d1.vd1.fa reference genome using each of the three aligners. The annotation file used for alignment was Gencode v28. HISAT2 and Magic-Blast were run with default parameters, while STAR was run in two-pass mode to enhance splice junction detection.

To standardize preprocessing across aligners, all BAM files underwent the following steps recommended by Mutect2: FixMateInformation, MarkDuplicates, SplitNCigarReads, BaseRecalibrator + ApplyBQSR. After preprocessing, variant calling was performed using MuTect2 (v4.0.5.2) across all aligners to ensure comparability. This workflow was designed to isolate the effects of alignment while ensuring consistent downstream variant calling and filtering criteria. To ensure a more reliable comparison across aligners, the final variant calls underwent stringent filtering: VAF filtering was adjusted per panel and aligner to achieve a FPR of 5 per million bases. Variants were required to have ALT DP ≥ 4 and total DP ≥ 20.

### Drug- or disease-related variant identification

The ClinVar annotation (2021-04) file was downloaded to support the query. SnpSift^29^ was used to obtain the clinical information with ClinVar information of the targeting variants. The clinical disease names (CLNDN) in the results were extracted to describe the functions of variants. Next, we predicted the potential impact of the variants via SnpEff. This tool provides a simple assessment of the putative variant impact on protein function, such as HIGH, MODERATE, or LOW impact.

## Supplementary information

Supplementary materials

Supplementary Table 5 List of RNA unique Variants and potential impacts

Supplementary Table 6 Details of KP variants

Supplementary Table 7 Information on libraries and panels

## Data Availability

All data generated or analyzed during this study are included in this published article and its supplementary information files.
