# Supplementary material for: Augmenting precision medicine via targeted RNA-Seq detection of expressed mutations
Source: NPJ Precis Oncol. 2025 Jun 13;9:182. doi: 10.1038/s41698-025-00993-8 (PMC12166063; doi:10.1038/s41698-025-00993-8)
Supplement: Supplementary file 1 — Supplementary materials [file 41698_2025_993_MOESM1_ESM.pdf]

Supplementary materials

Augmenting precision medicine via targeted RNA-Seq detection of expressed mutations

Dan Li, Jianying Li, Donald J. Johann Jr, Daniel Butler, Guangchun Chen, Jonathan Foox, Binsheng Gong, Wendell Jones, David P Kreil, Rebecca Kusko, Paweł P Łabaj, Anne Bergstrom Lucas, Christopher E Mason, Christopher Mozsary, Natalia Novoradovskaya, Carlos Pabón-Peña Pabon, Bohu Pan, Todd A. Richmond, Roberta Maestro, Sayed Mohammad Ebrahim Sahraeian, Andreas Scherer, Hagen U. Tilgner, James Willey, Pierre R. Bushel[†], Joshua Xu[†]

Supplementary Tables

Supplementary Table 1: Number of FP variant calls reported with non-stringent cutoff applied

**Targeted**	**Total**	**ADP=2**	**Mutect2**	**VarDict**	**LoFreq**
AGLR1-1	1925	1365	0	1925	0
AGLR1-2	1826	1284	2	1824	0
AGLR1-3	1958	1368	6	1952	0
AGLR1-4	1980	1392	1	1979	0
AGLR2-1	4246	3124	2	4243	1
AGLR2-2	4171	3091	4	4167	0
AGLR2-3	4313	3103	2	4311	0
AGLR2-4	3928	2878	1	3927	0
ROCR1-1	189	144	0	189	0
ROCR1-2	185	152	2	183	0
ROCR1-3	185	154	3	182	0
ROCR1-4	197	163	1	195	1
ROCR2-1	573	513	4	568	1
ROCR2-2	576	509	3	572	1
ROCR2-3	556	488	1	553	2
ROCR2-4	529	462	3	525	1
**WTS**	**Total**	**ADP=2**	**Mutect2**	**VarDict**	**LoFreq**
AGLR1-1	113	56	2	109	2
AGLR1-2	132	66	2	126	4
AGLR1-3	127	74	1	120	6
AGLR1-4	141	89	3	138	0
AGLR2-1	200	110	3	193	4
AGLR2-2	200	109	3	192	5
AGLR2-3	202	123	4	190	8
AGLR2-4	213	139	4	206	3
ROCR1-1	117	60	3	112	2
ROCR1-2	114	65	2	108	4
ROCR1-3	115	71	2	108	5
ROCR1-4	136	83	3	132	1
ROCR2-1	216	116	4	208	4
ROCR2-2	215	116	3	207	5
ROCR2-3	209	122	4	197	8
ROCR2-4	233	148	4	226	3

Using non-stringent cutoff as VAF ≥2%, total DP ≥20, alternative DP ≥2, reported by any caller (one out of three), a large number of FP calls were reported by the targeted RNA-seq data, especially in AGLR1 and AGLR2. We investigated the FP calls in detail and found that the majority of them had a low ADP of 2 and reported only by VarDict. In this table, we list the number of FP calls per replicate library and variant caller. To further control the FPR, we could require a higher ADP cutoff and a consensus call by multiple callers.

Supplementary Table 2: A cluster of FP calls located in proximity to one another

FP calls	VAF (rep1)	DP (rep1)	ADP (rep1)	In which Reps
chr3-147395919-A-G	0.126	175	22	1,2,3,4
chr3-147395925-A-G	0.198	172	34	1,2,3,4
chr3-147395930-A-G	0.090	134	12	4
chr3-147395987-A-G	0.080	113	9	1,4
chr3-147396152-A-G	0.069	87	6	1
chr3-147396195-A-G	0.121	66	8	1,2,3,4
chr3-147396201-A-G	0.115	61	7	1,2,3,4
chr3-147410769-A-G	0.104	125	13	1,2,3
chr3-147410868-A-G	0.202	84	17	1,2,4
chr3-147411011-A-G	0.065	278	18	3

In the ROCR1 panel, we identified a cluster of FP calls located in proximity to one another, which contributed to a heightened FPR in the VAF range above 5%. Some of the FP calls were consistently reported across multiple replicates. As shown in the table, these FP calls had low DPs and ADPs but still met our cutoffs.

Further investigations revealed that these calls were specific to the ROCR1 panel:

- All these ten positions were also covered by AGLR2 and ROCR2.
- None of them were detected by any DNA-seq panel that covered them.
- In targeted RNA-seq, two of them were detected by AGLR2 in a single replicate; four were detected by ROCR2 with two in only one replicate and the other two in three replicates.
- They were also detected in ROCR1 using different pipelines. HISAT2 + Mutect2 (eight calls), STAR + Mutect2 (nine calls), MagicBlast + Mutect2 (nine calls). Most of them were detected in multiple replicates.
- We also found all these ten calls in the WTS data for ROCR1.

Notably, all these calls were classified as "clustered_events" by Mutect2: Multiple events are present on the same haplotype as the variant which is indicative of a false-positive call. In conclusion, these calls were only seen in RNA-seq data, and enriched in ROCR1 panel even other panels covered these positions. This cluster of FP calls was excluded from all the panels for further analysis.

This range is the 3rd exon of the ZIC4 gene. Sequence analysis of the region surrounding these FP calls revealed a recurring motif where a string of adenine (A) nucleotides precedes a guanine (G) base.

Sequence (chr3:147395918-147396202):

A**A**GGACA**A**GGGA**A**GGGCTTCTCGCCCGTGTGCACGCGGATGTGATTTACAAGTTTGTATTTGGCTTTGA**A**GGGCTT
TCCCTGGCGCGGACACTCCTCCCAGAAGCAAATGTGGTTGGCCTGTTCCGGGCCGCCGACGTGCTCCACGGTGACG
TGCGTGACCAGCTCGTGCATGGTGCTGAAAGTTTTGGAGCAGAGGCTCGGGGTCGCGGTGCCGTCGGCCGCCAGC
CACTTGC**A**GATGAGCTCCTGTTTGATGGGCTGGCGCATGTAGCGGAAGAA**A**GCGCC**A**G

Supplementary Table 3: The targeted RNA-seq results of controlling the FPR to 5±1 per million bases

Targeted	VAF cutoff	ADP cutoff	FRP	KP variant	Recall	KP covered	Avg recall	Avg VAF cutoff
AGLR1-1	4.1%	4	4.46	1,115	40.7%	2,739	39.6%	4.4%
AGLR1-2	4.7%	4	5.57	1,090	39.8%			
AGLR1-3	4.3%	4	5.57	1,090	39.8%			
AGLR1-4	4.6%	4	5.57	1,043	38.1%			
AGLR2-1	4.1%	4	5.91	1,573	34.6%	4,548	34.9%	3.7%
AGLR2-2	3.9%	4	5.26	1,544	33.9%			
AGLR2-3	3.6%	4	5.91	1,575	34.6%			
AGLR2-4	3.0%	4	5.91	1,660	36.5%			
ROCR1-1	4.8%	4	4.76	1,121	42.0%	2,668	41.1%	5.2%
ROCR1-2	5.3%	4	5.95	1,098	41.2%			
ROCR1-3	5.0%	4	5.95	1,110	41.6%			
ROCR1-4	5.7%	4	5.95	1,056	39.6%			
ROCR2-1	6.5%	4	5.73	1,969	35.6%	5,527	37.3%	5.7%
ROCR2-2	5.0%	4	5.16	2,101	38.0%			
ROCR2-3	6.0%	4	4.59	2,045	37.0%			
ROCR2-4	5.3%	4	5.73	2,077	37.6%			

Starting with VAF =2%, ADP =4, DP =20, and detection by at least two callers, we increased the VAF cutoff by 0.1% to control the FPR around 5 per million bases. For each panel, we observed similar numbers across replicates, and the average of recall and VAF cutoff were calculated.

Supplementary Table 4: The WTS results of controlling the FPR to 5±1 per million bases

WTS	VAF cutoff	ADP cutoff	FRP	KP variant	Recall	KP covered	Avg recall	Avg VAF cutoff
AGLR1-1	9.6%	4	5.57	970	35.4%	2,739	35.3%	9.275%
AGLR1-2	7.4%	4	5.57	1,026	37.5%			
AGLR1-3	10%	4	5.57	948	34.6%			
AGLR1-4	10.1%	4	5.57	924	33.7%			
AGLR2-1	9.5%	4	5.26	1,492	32.8%	4,548	32.65%	9.3%
AGLR2-2	7.4%	4	5.91	1,599	35.2%			
AGLR2-3	10.2%	4	5.91	1,447	31.8%			
AGLR2-4	10.1%	4	5.91	1,403	30.8%			
ROCR1-1	9.6%	4	5.95	787	29.5%	2,668	29.325%	9.5%
ROCR1-2	8.2%	4	5.95	820	30.7%			
ROCR1-3	10%	4	5.95	781	29.3%			
ROCR1-4	10.2%	4	5.95	742	27.8%			
ROCR2-1	9%	4	5.73	1,785	32.3%	5,527	31.75%	8.95%
ROCR2-2	7.4%	4	5.16	1,847	33.4%			
ROCR2-3	10%	4	5.73	1,705	30.8%			
ROCR2-4	9.4%	4	5.73	1,687	30.5%			

Starting with VAF =2%, ADP =4, DP =20, and detection by at least two callers, we increased the VAF cutoff by 0.1% to control the FPR around 5 per million bases. To achieve the FPR as 5 per million bases, much higher VAF cutoffs were required by the WTS data, resulting in fewer KP variants being detected and about 10% lower recall compared to the targeted RNA-seq panels. Interestingly, WTS replicate 2 appeared to perform better than other replicates. We restricted the same WTS results to different panels regions to obtain WTS-panel results, so that all the replicate 2 of panels performed better than others.

Supplementary Table 5: List of RNA unique Variants and potential impacts
Please see the "Supplementary Table 5 - List of RNA unique Variants and potential impacts.xlsx" file.

Supplementary Table 6: Details of KP variants
Please see the "Supplementary Table 6 - Details of KP variants.xlsx" file.

Supplementary Table 7: Information on libraries and panels
Please see the "Supplementary Table 7 - Information on libraries and panels.xlsx" file.

Supplementary Figures

A

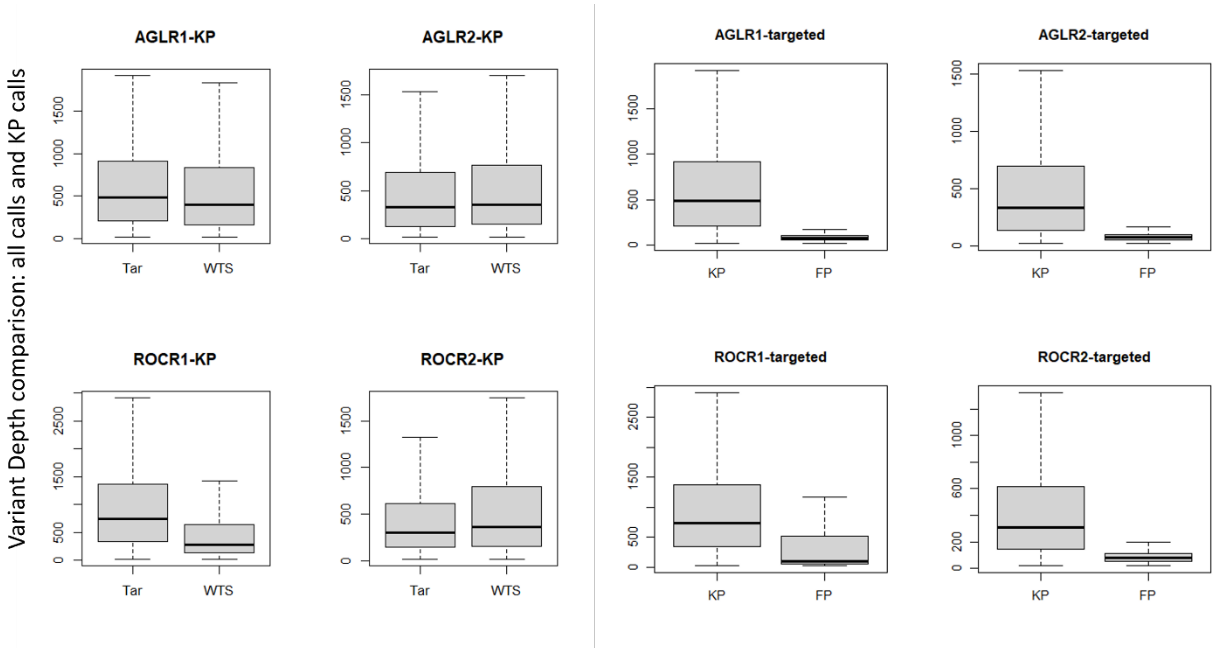

B

Panel	**KP covered**	**Detected (Targeted)**	**Detected (WTS)**	**#KP (Targeted)**	**#KP (WTS)**	**Targeted #FP**	**WTS #FP**
AGLR1	2739	5407.5	2176.25	1728.25 (63.1%)	1698.25 (62.0%)	1941.5	180.5
AGLR2	4548	9859.25	3204.5	2332.25 (51.3%)	2506.75 (55.1%)	4185	277.5
ROCR1	2668	2125.75	1772	1572 (58.9%)	1360 (51.0%)	233.25	171.25
ROCR2	5527	4366	3740.25	2978 (53.9%)	2962.75 (53.6%)	621	298

Supplementary Figure 1. Comparison between Targeted RNA-seq and WTS results with non-stringent cutoff applied to control the FPR. We set the non-stringent cutoff as VAF ≥2%, total DP ≥20, alternative DP ≥2, and reported by any of the three callers. **A)** The WTS data achieved similar recall levels in all the penal regions compared to the targeted RNA-seq, with fewer total and FP calls reported. We then investigated the variants depth between various groups of calls using replicate1 as an example. For the KP variants, the variant depths were similar between targeted and WTS (the ROCR1 panel was extremely deep). The depths of KP variants in WTS were similar (median DP = 396, 358, 284, 366.5). The DPs of FP calls in targeted RNA-seq results were significantly lower compared to KP variants. **B)** Without controlling the FPR, many calls with weak signals could be picked up by the computational pipelines.

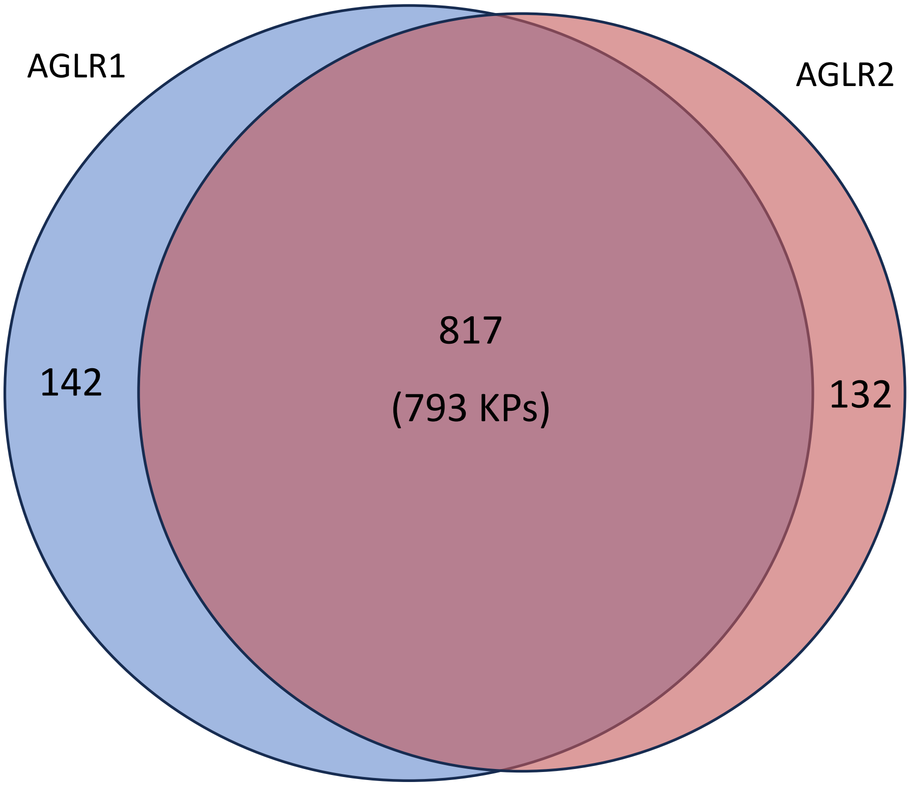

Supplementary Figure 2. In the overlap regions, the overlap rates of AGLR1 and AGLR2 were 85.2% and 86.1%, respectively. Notably, the reproducibility (across replicates) of AGLR1 and AGLR2 was approximately 87-88%. The results in the overlap regions of these two panels were consistent. The results were obtained after the FPR was reduced to 5 per million bases.

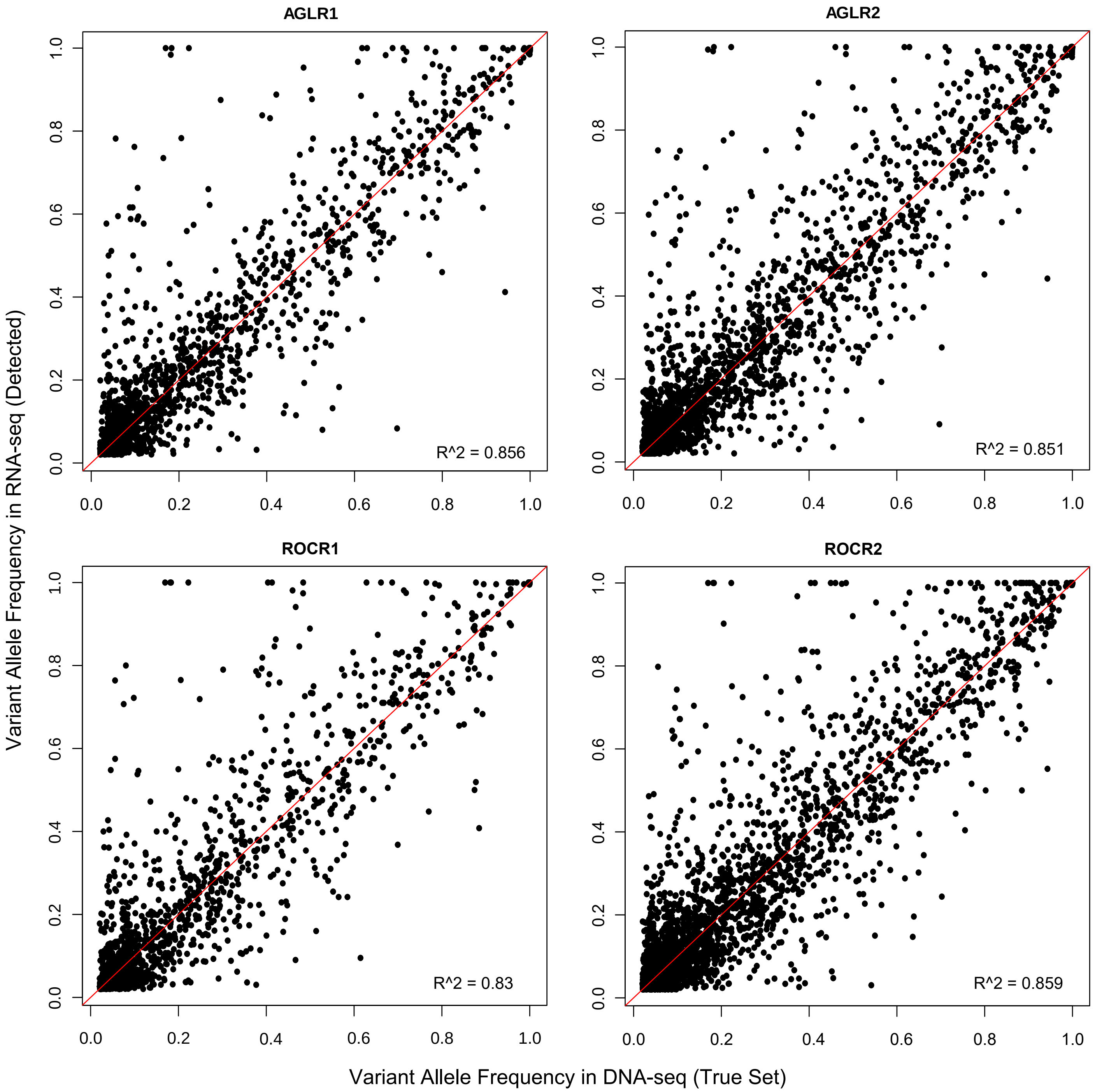

Supplementary Figure 3. Comparison of variant allele frequencies (VAFs) between DNA-seq (true set) and RNA-seq (detected variants) across the four targeted panels (AGLR1, AGLR2, ROCR1, and ROCR2). Each scatter plot represents the relationship between true VAFs from DNA-seq (x-axis) and the VAFs detected in RNA-seq (y-axis) for a given panel. A strong correlation is observed across all panels, with R^2 values consistently around 0.85, indicating good agreement between RNA- and DNA-derived VAFs. Some variations are expected due to differences in gene expression, allele-specific expression, and RNA processing effects. The high correlation supports the reliability of RNA-seq for detecting expressed variants.

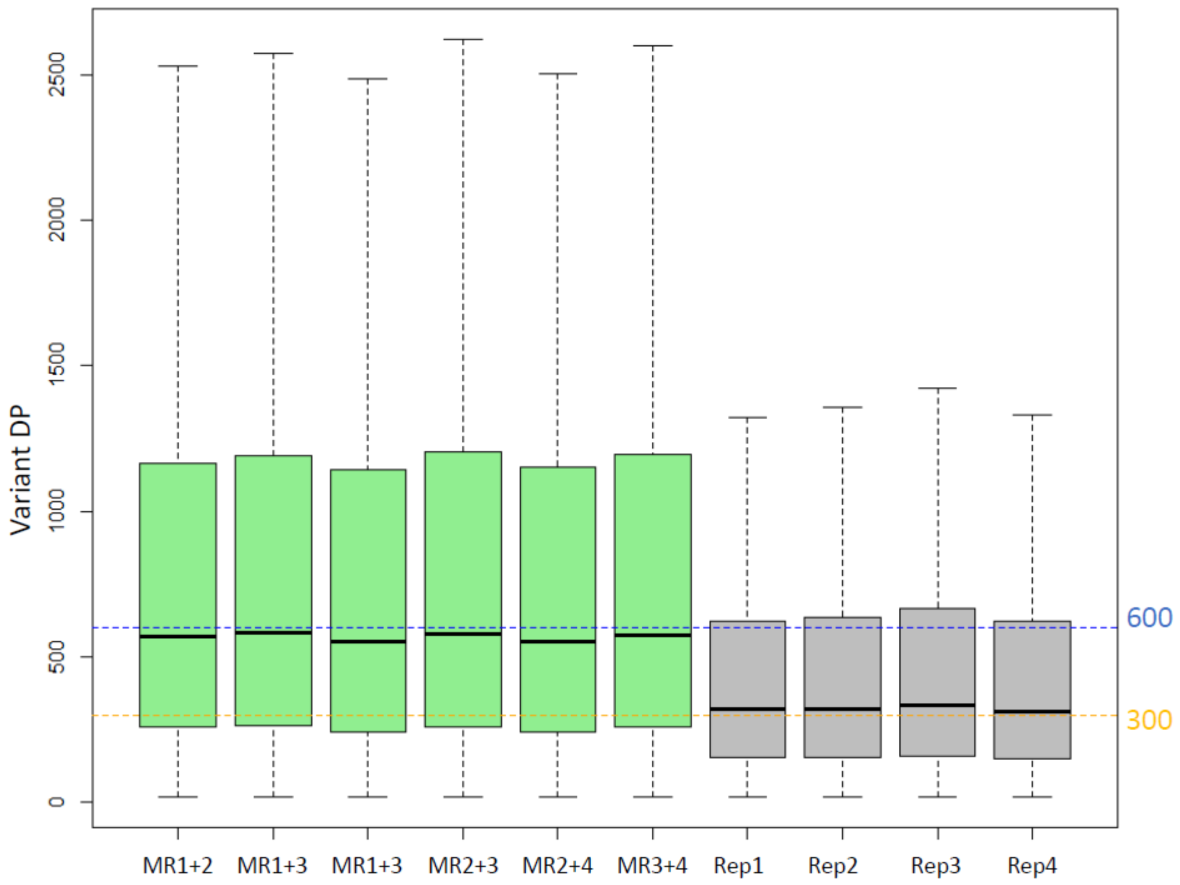

Supplementary Figure 4. The variant DP comparison between merged-libraries and single-libraries of the ROCR2 panel. The merged libraries were created by combining the alignment files of two replicate libraries together. On average, the median variant DP of merged-libraries was twice as much as single-libraries.

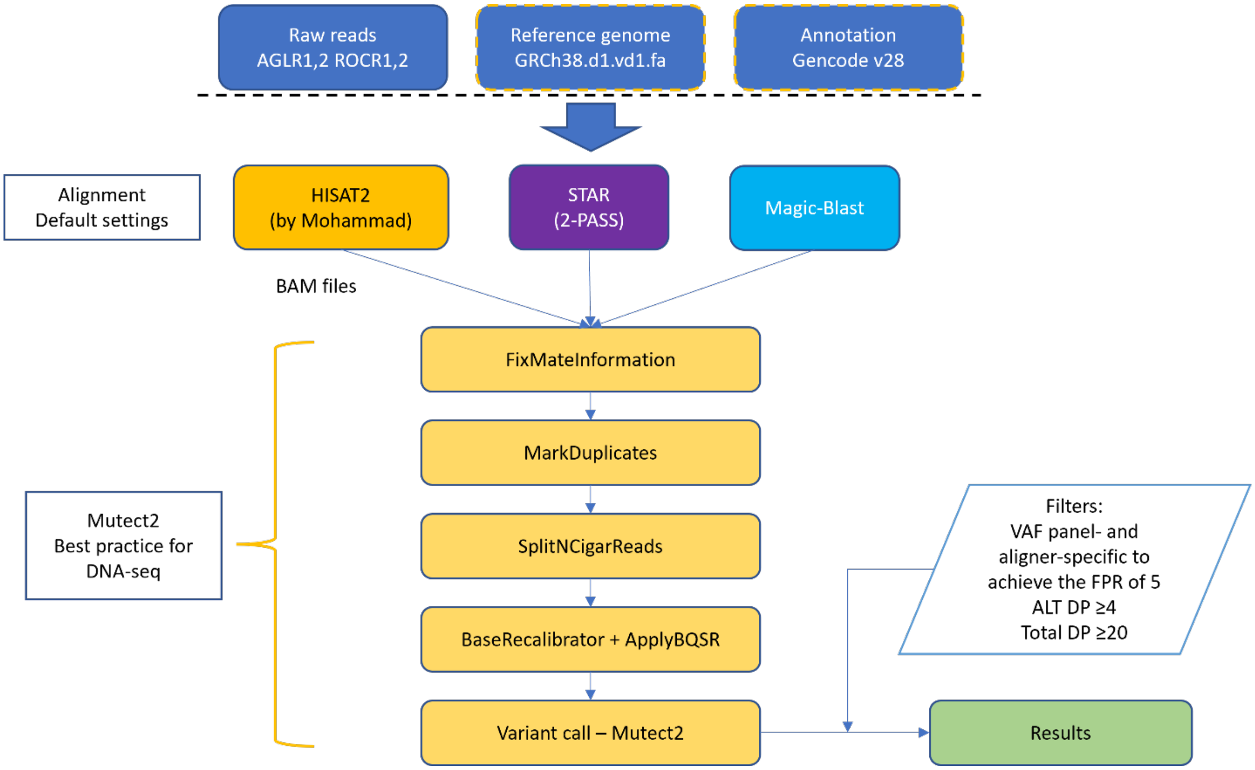

Supplementary Figure 5. The workflow of aligner comparison on RNA-seq variant detection. Three aligners HISAT2, STAR, and Magic-Blast were used to map the RNA-seq data of each panel to the reference genome (GRCh38.d1.vd1.fa). Gencodev28 gene annotation file was applied. With the default parameter settings, BAM files were generated and then fed to Mutect2 for variant calling. Identical procedures were followed for a solid comparison. Additional cutoffs were added at the end to obtain high-confident variants.

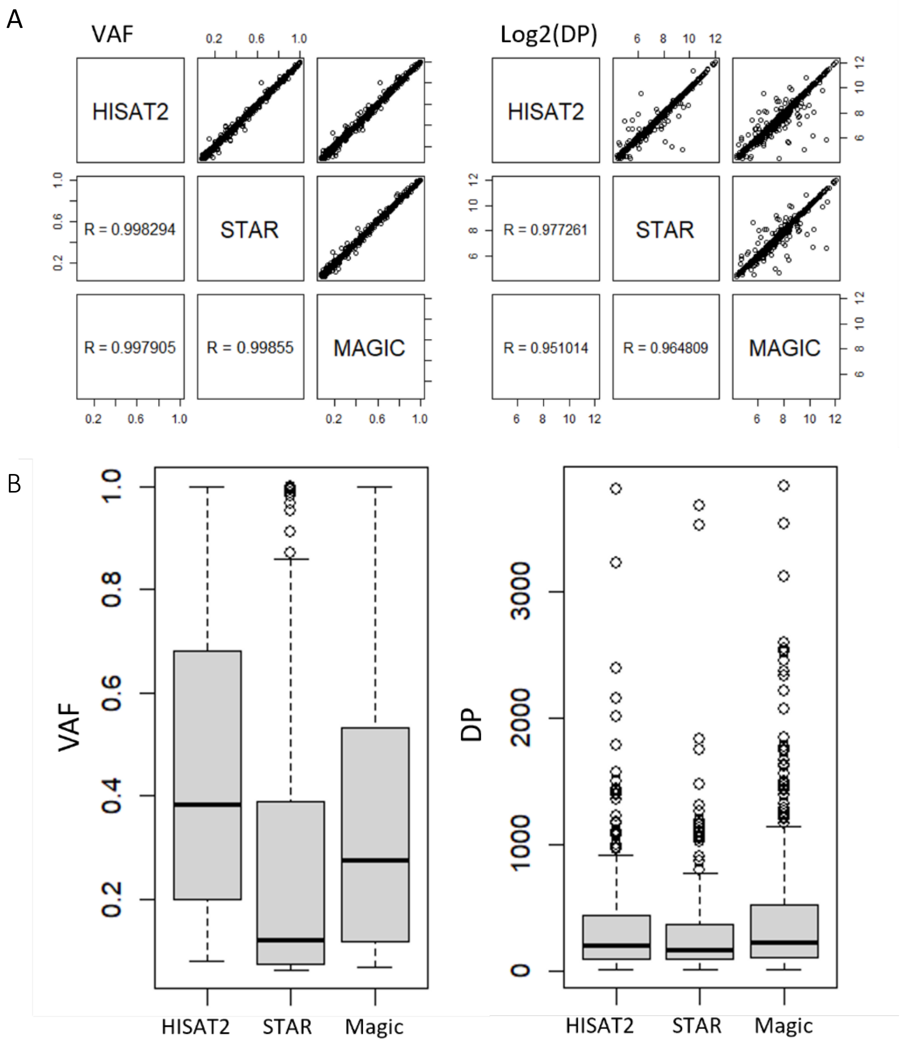

Supplementary Figure 6. Comparison of variant detection across pipelines based on various aligners (ROCR2 replicate 1 as an example). **A**) The comparison of VAF and log2(DP) of the variants shared by the three aligners. Similar values were observed, resulting in very high R-squared values. **B**) We also investigated the VAFs and DPs of the unique variants per aligners. However, no significant difference was observed. The differences may be due to some technical settings of the aligners.

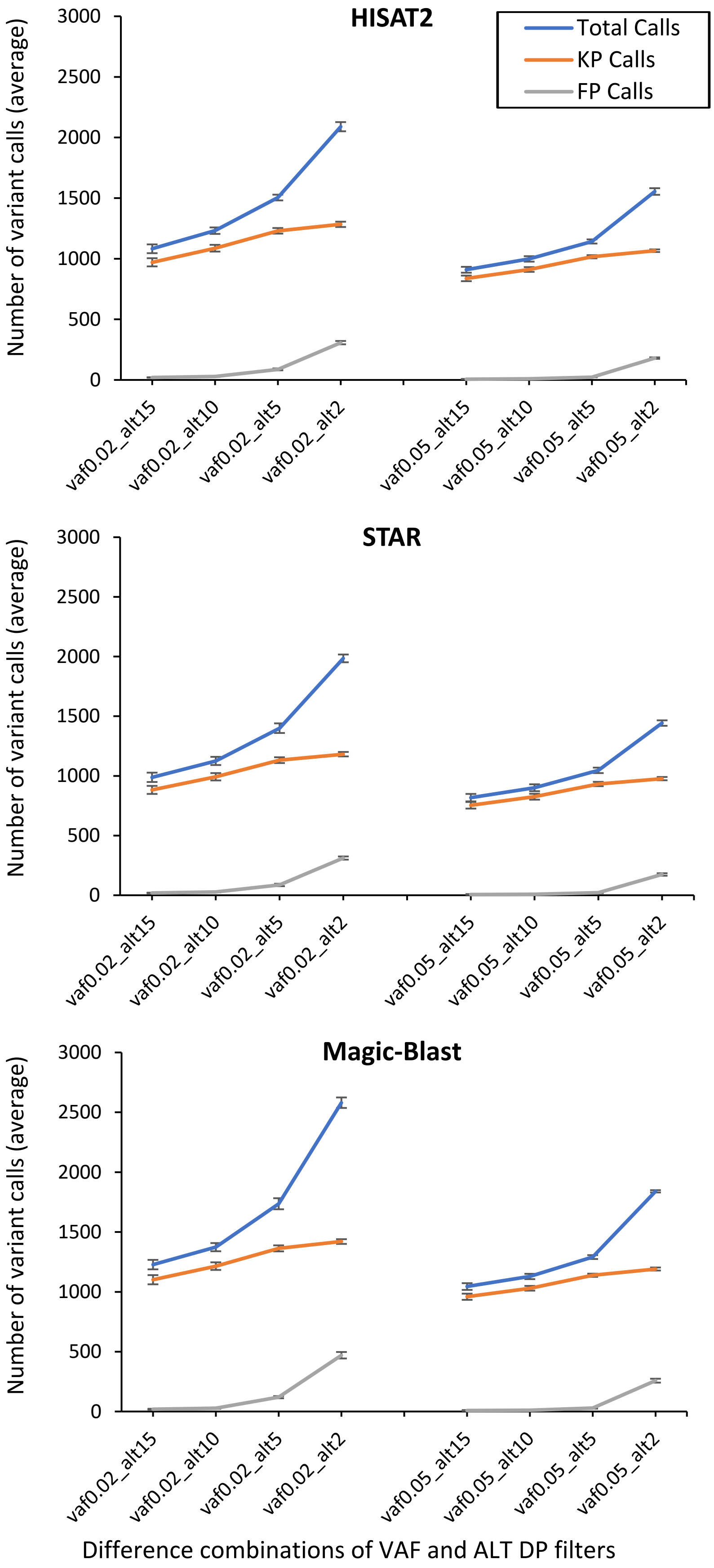

Supplementary Figure 7. Impact of filtering parameters (VAF and ALT DP) on variant detection across different aligners. The number of variant calls (average across replicates) is shown for AGLR1 panel using three different aligners. Each plot displays the total number of variant calls (blue line), known positive (KP) variant calls (orange line), and false positive (FP) calls (gray line) under varying filter thresholds. The x-axis represents different filtering conditions based on VAF and LT DP. The error bars represent standard deviations across replicates, indicating variability in variant detection under different filtering criteria.

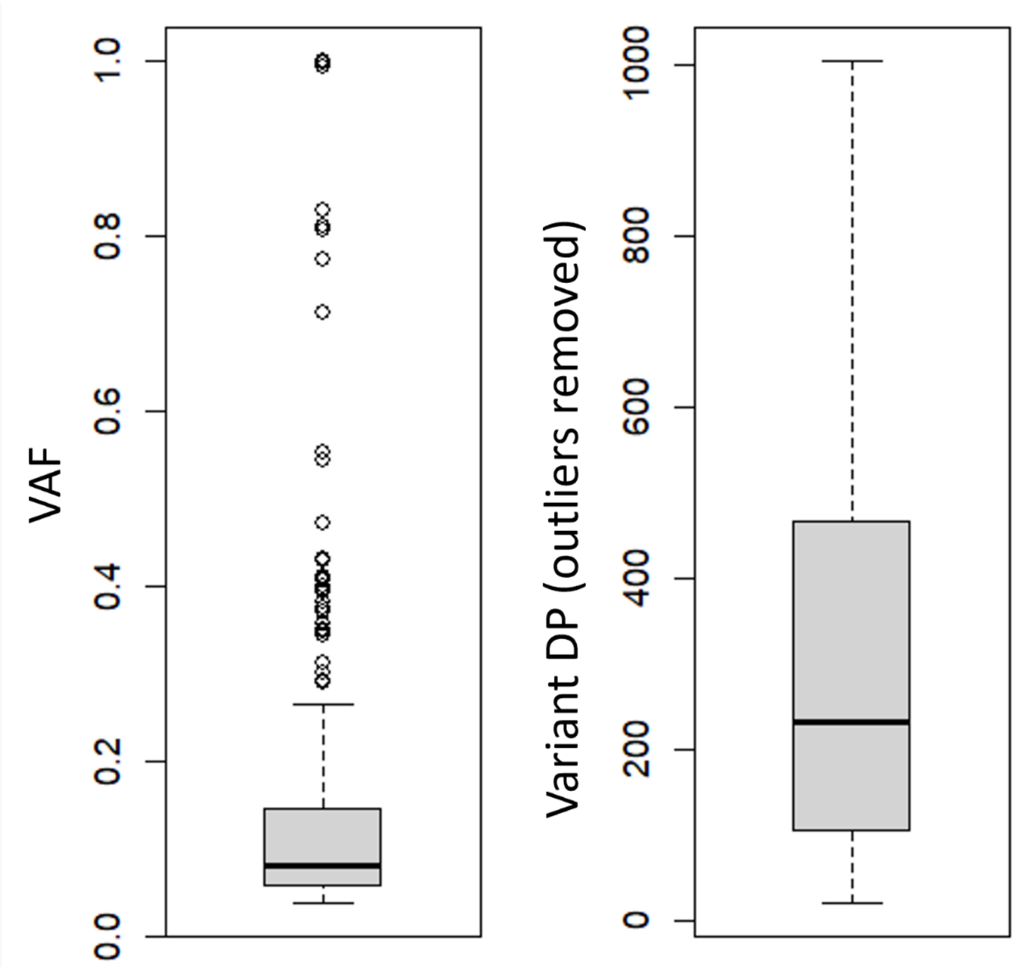

Supplementary Figure 8. The distributions of VAF and DP for the RNA-unique variants. The boxplots included all RNA-unique variants detected in different panels. These variants had high VAFs and DPs that met the cutoffs specified for each panel for the purpose of controlling the FPR.

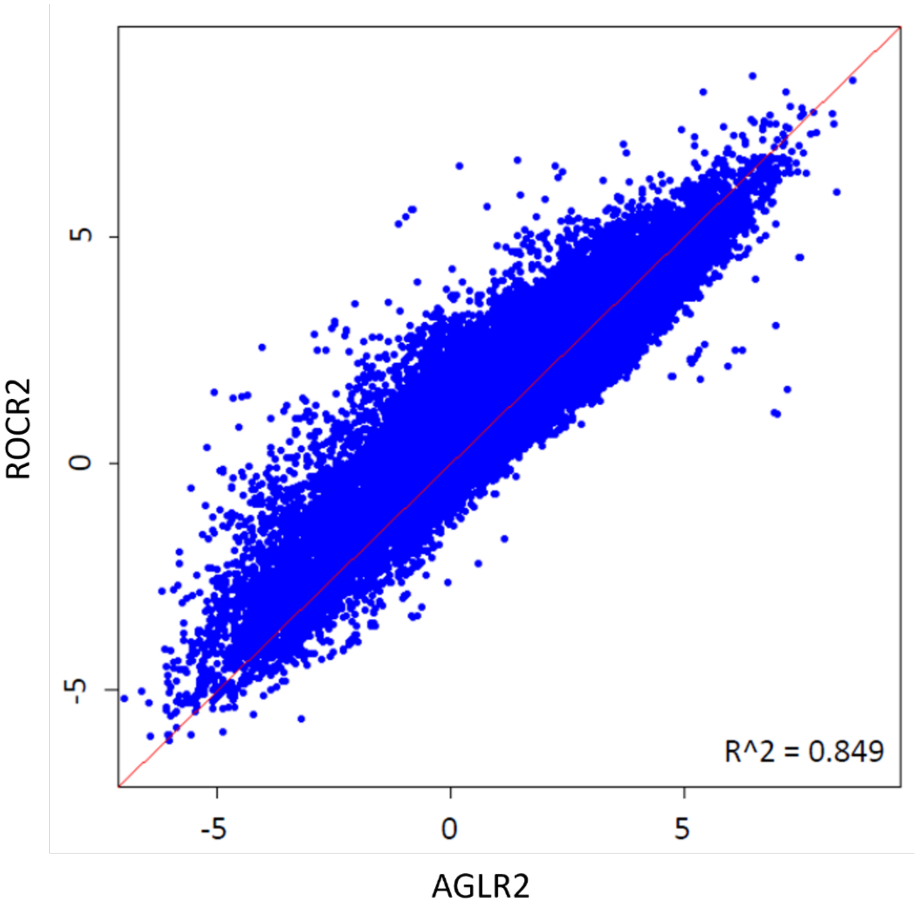

Supplementary Figure 9. Expression comparison of 36,531 exons overlapping with the intersection regions of AGLR2 and ROCR2 in CTR. The expression, calculated as the log2 of the average transcript per million (TPM) across four replicates, showed that exon expressions reported by the two panels were comparable.

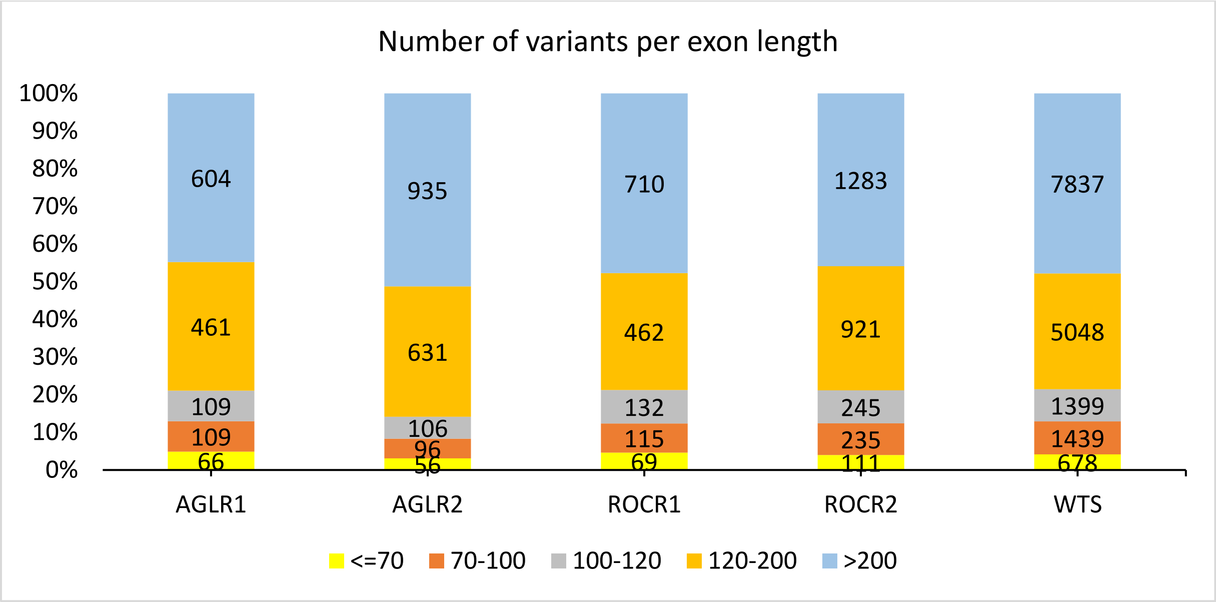

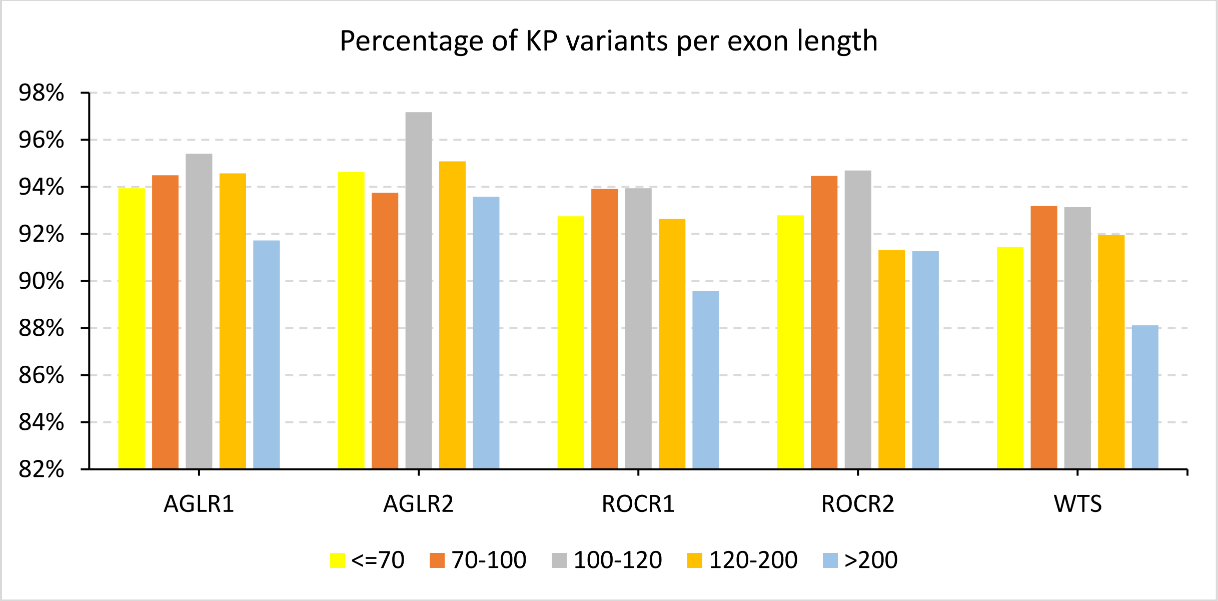

Supplementary Figure 10. We grouped the exons that cover the variants based on their lengths. A, The distribution of the number of variants per exon length. Certainly, more variants were covered by longer exons, and the percentages were similar across panels. AGLR2 panel was slightly different from others that less (in percentage) variants were detected in smaller exons (≤120bps). B, Percentage of the KP variants per exon length. The longer exons (>120) covered more non-KP variants in ROCR (probe length 75~100) and WTS panels than in AGLR (probe length 120) panels.
